# A systems biology-based mathematical model demonstrates the potential anti-stress effectiveness of a multi-nutrient botanical formulation

**DOI:** 10.1038/s41598-024-60112-8

**Published:** 2024-04-26

**Authors:** Abha Saxena, Kaushiki S. Prabhudesai, Aparna Damle, Shyam Ramakrishnan, Palaniyamma Durairaj, Sumathi Kalankariyan, A. B. Vijayalakshmi, K. V. Venkatesh

**Affiliations:** 1 MetFlux Research Private Limited, Bengaluru, India; 2 Amway Global Services India Pvt. Ltd., Gurugram, India; 3 Amway Corporation, Ada, USA; 4 Department of Chemical Engineering, Indian Institute of Technology Bombay, Mumbai, Maharashtra India

**Keywords:** Computational biology and bioinformatics, Systems biology, Biomarkers, Health care, Mathematics and computing

## Abstract

Stress is an adaptive response to the stressors that adversely affects physiological and psychological health. Stress elicits HPA axis activation, resulting in cortisol release, ultimately contributing to oxidative, inflammatory, physiological and mental stress. Nutritional supplementations with antioxidant, anti-inflammatory, and stress-relieving properties are among widely preferred complementary approaches for the stress management. However, there is limited research on the potential combined impact of vitamins, minerals and natural ingredients on stress. In the present study, we have investigated the effect of a multi-nutrient botanical formulation, Nutrilite^®^ Daily Plus, on clinical stress parameters. The stress-modulatory effects were quantified at population level using a customized sub-clinical inflammation mathematical model. The model suggested that combined intervention of botanical and micronutrients lead to significant decline in physical stress (75% decline), mental stress (70% decline), oxidative stress (55% decline) and inflammatory stress (75% decline) as evident from reduction in key stress parameters such as ROS, TNF-α, blood pressure, cortisol levels and PSS scores at both individual and population levels. Further, at the population level, the intervention relieved stress in 85% of individuals who moved towards a healthy state. The in silico studies strongly predicts the use of Gotukola based Nutrilite^®^ Daily Plus as promising anti-stress formulation.

## Introduction

Stress can manifest itself in various daily challenges, including those related to physical work, mental health, and immune system suppression. The activation of hypothalamic–pituitary–adrenal (HPA) axis in response to environmental stressors results in excess release of cortisol, which is functional in linking chronic stress, anxiety, and depression with physical stress^1^. Most stress conditions are also caused by increased oxidative stress and generation of reactive oxygen species (ROS). The physiological disturbances in the redox status of biological molecules caused by oxidative stress and ROS have been closely associated with a variety of detrimental pathological conditions. Altered redox status stimulates major molecular pathways like nuclear factor kappa B (NF-κB), protein kinase C (PKC), phosphoinositide 3-kinase (PI3K), nuclear factor erythroid 2-related factor 2 (Nrf2), mitogen-activated protein kinase (MAPK), and production of various cytokines, including interleukin (IL)-1ß, IL-6, tumour necrosis factor-α (TNF-α) subsequently elevating systemic stress^2^. This can further induce serine phosphorylation of IRS (pIRS) and AKT (pAKT) modulating downstream signalling processes^3,4^. Antioxidant enzymes, including superoxide dismutase (SOD), catalase (CAT), and glutathione peroxidase (GPx), may protect against the harmful effects of ROS^5^.

Recently, there has been a growing interest in identifying natural ingredients with antioxidant properties, as they are considered to be safer and more acceptable to consumers^6^. In light of this, plant extracts and micronutrients that possess antioxidant properties may be employed as complementary or alternative approach to impede or prevent the development of comorbidities. Medicinal plants are rich source of essential metabolites and have potential antioxidant, antimicrobial, anti-inflammatory and stress-relieving activities^7^. Four such medicinal plants including Gotukola (*Centella asiatica* (L.))^8–12^, acerola cherry (*Malpighia emarginata* DC)^13,14^, elderberry (*Sambucus* spp.)^15–17^ and Purple carrots (*Daucus carota* L.)^18–20^ have been shown to possess stress-relieving property by targeting nodal molecular points such as NF-κB, TNF-α, IL-1, IL-6, Nrf2 and SOD.

Micronutrients including vitamins A, D and K aid in the reduction of pro-inflammatory cytokines such as NF-κB, TNF-α, IL-6, and IL-1β^21–23^. Similarly, vitamin C and E supplementation improved mental health as well as inflammatory parameters^24–26^. Several studies have reported that B vitamins supplementation exerts anti-inflammatory, anti-depressant and anti-oxidative effects by suppressing pro-inflammatory cytokines, elevating expression of anti-oxidant enzymes and regulating cortisol levels^27–36^. Likewise, studies have demonstrated anti-oxidant, anti-inflammatory and anti-depressant properties of various minerals including calcium, iron, zinc, selenium, manganese and chromium by lowering levels of ROS, IL-6, TNF-α and NF-κB activity^37–42^. Iodine supplementation effects were represented by correlations with several parameters associated with stress conditions such as GPx, SOD, CAT, or plasma levels of IL-6^40^. Another study focused on plasma Adrenocorticotropic hormone (ACTH), IL-6, and cortisol levels to determine if magnesium supplementation might reduce damaging stress effects^43^. The effects of copper and molybdenum on stress and their correlation with cortisol concentrations have also been studied^44^.

Although several studies provide information regarding the individual effect of botanicals and micronutrients on clinical biomarkers, there is insufficient data on anti-stress effect of these compounds when used in combination. Thus, the current study aims to bridge the gap by assessing the combined role of a formulation containing vitamins, minerals, and botanical extracts on stress variables to improve clinical stress related parameters affecting an individual’s health. Systems biology-based mathematical modelling and simulation studies enable integration and provide fundamental insights into the mechanisms underlying various types of stress developed as a result of an imbalance in the homeostatic state. The current study extends these findings through quantitative analysis using mathematical modelling simulations, validation and targeting the biological markers related to physical, mental, oxidative, and inflammatory stress parameters to evaluate the stress-relieving effects of Nutrilite^®^ Daily Plus, a formulation containing botanicals including Gotukola, acerola cherry, elderberry, and purple carrot, along with essential vitamins and minerals. It was hypothesized that the combination of botanical extracts with micronutrients would improve clinical parameters, mood symptoms, and eventually, stress-related outcomes.

## Results

**Figure 1:**
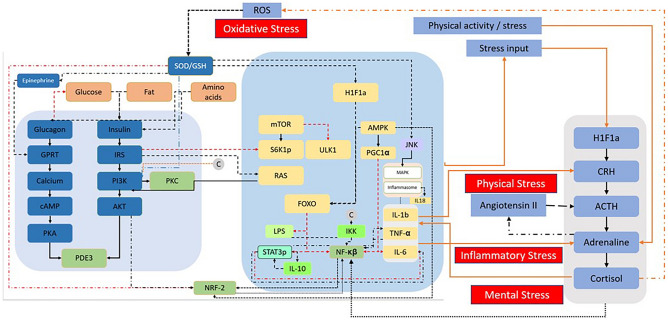
A comprehensive simulation model depicting the evaluation of synergistic impact of mixture of botanical extracts and micronutrients intervention on oxidative, inflammatory, physical, and mental stress at individual and population levels under various stress conditions towards achieving a healthy state.

The intervention effect is simulated, and results are analysed for oxidative, inflammatory, physical, and mental stress at an individual and population level. The impact of intervention has been tested under different stressed conditions and evaluated for the remission of the increased levels towards a healthy state. Towards this a systems biology based mathematical model is built using the pathways incorporated in various types of stress including oxidative, inflammatory, physical, and mental stress. The key nodes of the pathways, such as NF-κB and NRF2, are tweaked to induce a stress condition, confirmed by altered levels of stress markers such as ROS, TNF-α, Systolic blood pressure (SBP), and cortisol (Perceived stress scale (PSS)) (Fig. 1, more details provided in subsection model description under methods section).

Various molecular targets, such as cortisol, SOD, TNF-α, and ROS production percentage, are benchmarked with respect to the literature data to reflect similar dynamics between healthy and stressed conditions (Figure S1). Along with these pIRS/IRS and pAKT/AKT ratios were also benchmarked to the model due to their association with oxidative stress and ER stress respectively^3,4^. Further, the accuracy and efficiency of this subclinical inflammatory model is assessed by comparing the results reported in literature when supplemented with botanicals and micronutrients under consideration. When literature-based data is simulated using the model, fold change values of TNF-α, SBP, ROS and IL-6 obtained post intervention are observed to be in par with that demonstrated in the literature, confirming the validity of the model (Figure S2 a-d, File S1, Table S1).

### Steady-state dynamics with intervention effect

**Figure 2:**
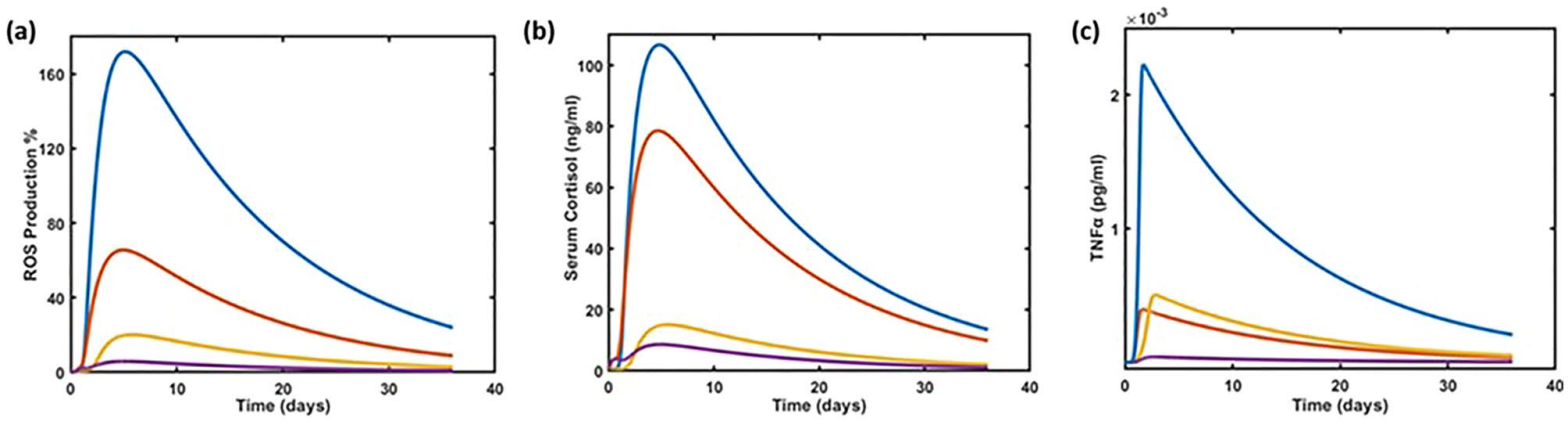
Steady-state dynamics comparing intervention effect on clinical stress parameters. (**a**) ROS production, (**b**) Cortisol levels, and (**c**) TNF- α levels. Plot representation (Blue: Under stress, without intervention; Red: Under Stress, only with micronutrients; Yellow: Under stress, only with mixture of botanical extracts; Purple: Under stress, with combination of botanical extracts and micronutrients).

The effect of the intervention is compared for steady-state levels of clinical stress parameters defining oxidative, inflammatory, physical, and mental stress. The model is configured to decipher the parameters that are highly responsive to the perturbation of a network under stressed environment (Fig. 1). The enhanced steady-state levels of clinical biomarkers of stress including ROS, cortisol, and TNF-α levels as compared to healthy individuals confirmed the implementation of stressed condition (Figure S3 a–c). The alteration in the steady-state levels of ROS, cortisol, and TNF-α are monitored when administered with mixture of botanical extracts and micronutrients individually as well as in combination (Fig. 2).

Compared to the stressed individuals, a significant decline is observed in the peak values of ROS production due to intervention (Fig. 2a). The oxidative stress peak value of ROS production is reduced from 171.92 to 5.58% (decreased by 30.8-fold) with the effect of the Nutrilite^®^ Daily Plus. Mental stress, defined by the PSS score, is associated with cortisol levels (Fig. 2b), having a peak value of 106.7 ng/mL during stress. This value is decreased by 13-fold, resulting in lowered peak value of 8.57 ng/mL. The formulation also has a beneficial impact on TNF-α levels associated with inflammatory stress and physical stress. The peak value of 2.2 × 10^−3^ pg/mL during stress is reduced by 53 times, decreasing it to 4.1 × 10^–5^ pg/mL with product intervention (Fig. 2c).

### Population analysis

The stress-mitigating property of botanicals, micronutrients, and their combination is further analysed using in silico adult population consisting of both males and females in equal ratio (age range: 20–80 years). The simulation results show that under stress-induced conditions, approximately 90% of the individuals are classified as highly stressed, with elevated ROS, TNF-α, and cortisol levels.

**Figure 3:**
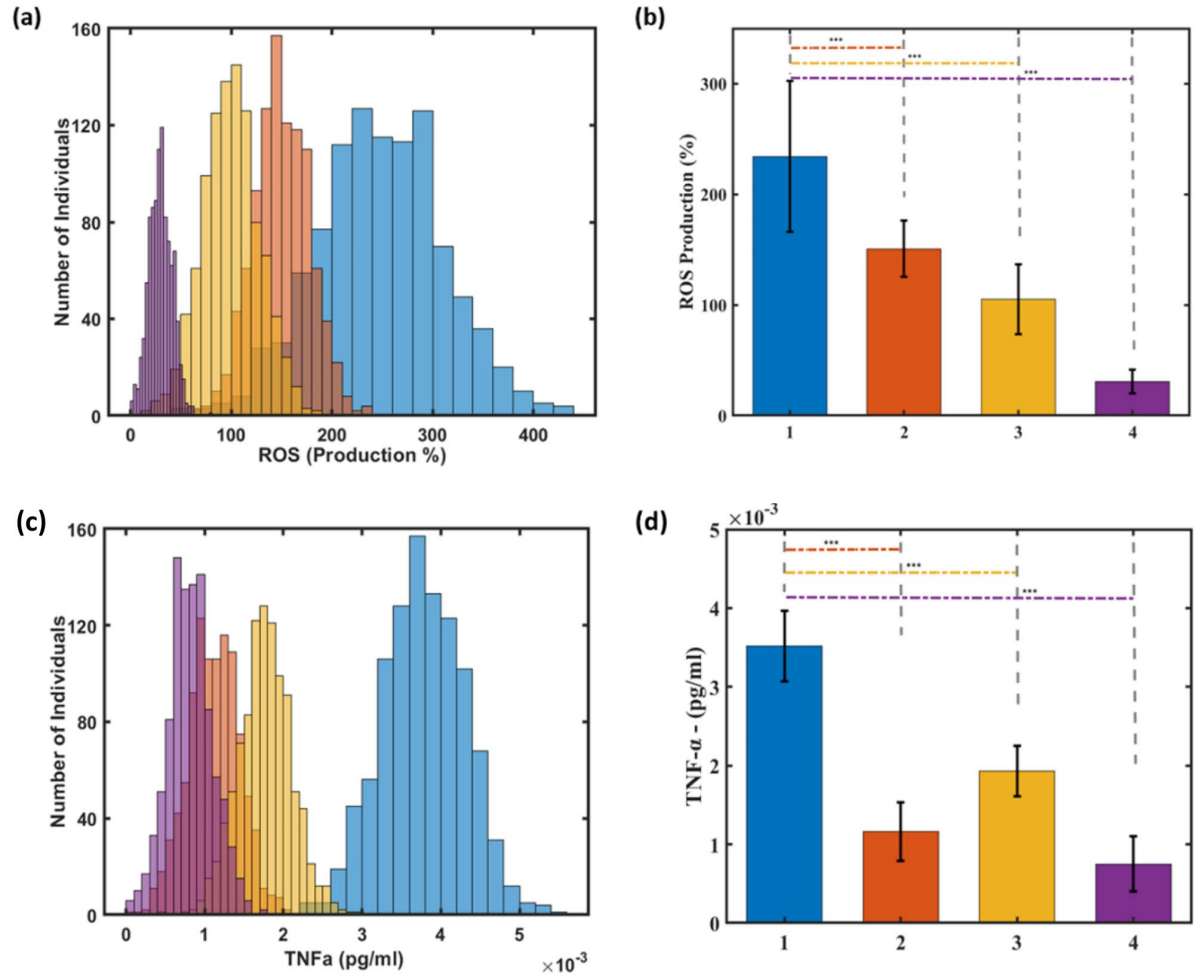
Population analysis comparing intervention effect on ROS production and TNF-α levels for oxidative and inflammatory stress respectively. (**a**) Population dynamics for change in ROS production, (**b**) Mean change in % ROS production (**c**) Population dynamics for change in TNF-α levels. (**d**) Mean change in TNF-α levels. Plot representation (Blue: Under stress, without intervention; Red: Under Stress, only with micronutrients; Yellow: Under stress, only with mixture of botanical extracts; Purple: Under stress, with combination of botanical extracts and micronutrients). Student’s t-test indicates ***p < 0.005.

When provided with micronutrients or mixture of botanical extracts in isolation, 40% and 55% of individuals show a decline in oxidative stress, respectively (Fig. 3a, Table S2). Figure 3b shows a significant reduction (*p*-value < 0.005) in the mean value of ROS production, lowering it from 248.42 (± 62.98)% to 29.92 (± 11.25)%, within 85% of the stressed population when provided with combination of botanical extracts and micronutrients. Similarly, TNF-α levels decreased significantly (*p*-value < 0.005) in 75% of the individuals subjected to the combination effect (Fig. 3c, Table S2). Figure 3d shows that the formulation decreases the stressed mean values from 3.8 (± 0.52) × 10^−3^ pg/mL to 0. 8 (± 0.29) × 10^−3^ pg/mL, lowering inflammatory stress.

**Figure 4:**
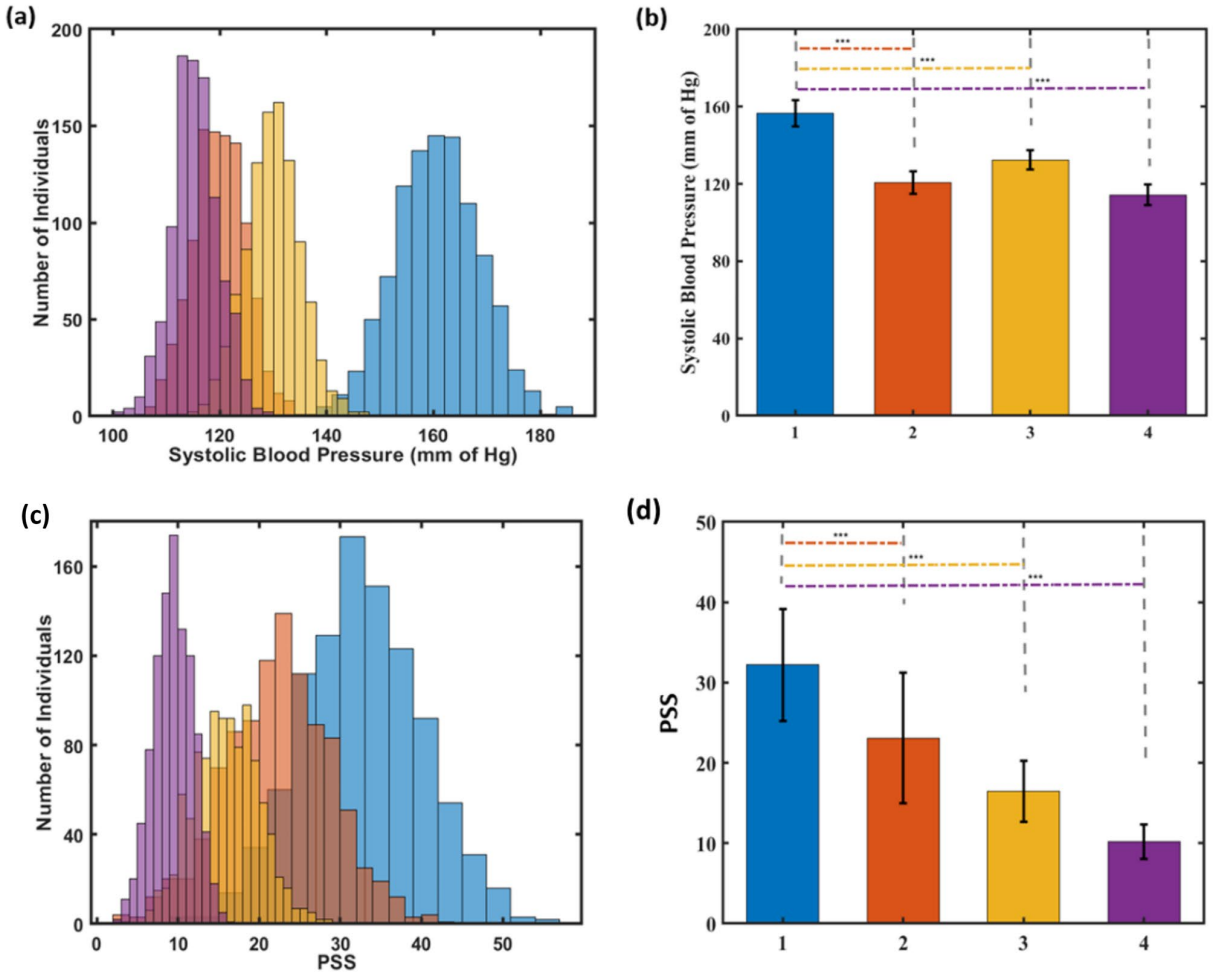
Population analysis comparing intervention effect on SBP and PSS for physical and mental stress respectively. (**a**) Population dynamics for change in SBP, (**b**) Mean change in SBP levels (**c**) Population dynamics for change in PSS. (**d**) Mean change in PSS. Plot representation (Blue: Under stress, without intervention; Red: Under Stress, only with micronutrients; Yellow: Under stress, only with mixture of botanical extracts; Purple: Under stress, with combination of botanical extracts and micronutrients). Student’s t-test indicates ***p < 0.005.

While investigating the physical stress trends within the stressed population, it is observed that 98% of the individuals have a high BP range above 120 mm Hg (Fig. 4a, Table S2). The stress reduction results showed greater improvement with the use of either the micronutrient or its combination with botanical extracts, as compared to the population that only used the botanical extracts in the simulation. Figure 4b shows that combination of both micronutrients and mixture of botanical extracts contributed to a significant decline (*p*-value < 0.005) in BP, bringing it down from 160.71 (± 7.92) mm of Hg to 115.41 (± 4.33) mm of Hg, which is the normal range for BP levels. When analyzing the mental stress parameter for the stressed population, 98% of the individuals have elevated stress values, having a PSS score of more than 13. Figure 4c shows intervention with micronutrients and botanicals combination reduces the mental stress for 85% of the individuals. The reduction in the mental stress is highly significant (*p*-value < 0.005), as indicated by the mean PSS score decreasing from 32.72 (± 8.45) to 9.04 (± 3.38), which falls within the low-stress range of the PSS score (Fig. 4d, Table S2).

The effectiveness of the combined formulation is further assessed by ascertaining the percentage of individuals whose biomarker levels (% ROS, TNF-α, SBP and PSS) fell below the typical mean biomarker levels found in individuals with stress. With respect to oxidative stress marker, it is observed that in 99% individuals, ROS levels are lower than mean ROS levels in stress condition. Approximately 98.9% of the individuals with oxidative stress have fewer chances of having stress symptoms, and they demonstrate remittance towards the healthy/mild range of ROS production levels, i.e., 0–10% species (Figure S4a). All the individuals belonging to the combined intervention group have TNF-α values lower than mean values observed in stress condition indicating its 100% effectiveness. Interestingly, 99.2% of population has TNF-α levels falling within the healthy range, indicating complete remission of inflammatory stress (Figure S4b). Correspondingly, the combination is successful in reducing the levels of SBP to the normal levels in 98.7% of the population suggestive of remission of physical stress in these individuals whereas, all 100% individuals have SBP levels below mean levels noted for stressed condition (Figure S4c). The similar beneficial effect is observed on mental stress. The combination is effective on 99% of the population, with 97.6% showing complete remission for mental stress as PSS scores ranged from healthy to mild stress values of 0–13 (Figure S4d).

Based on the population analysis, the combined effect of botanicals extract and micronutrient is more profound as observed from a significant reduction in stress through clinical markers like ROS, TNF-α, BP, and PSS scores representing oxidative, inflammatory, physical, and mental stress.

### Stress phenotype analysis

**Figure 5:**
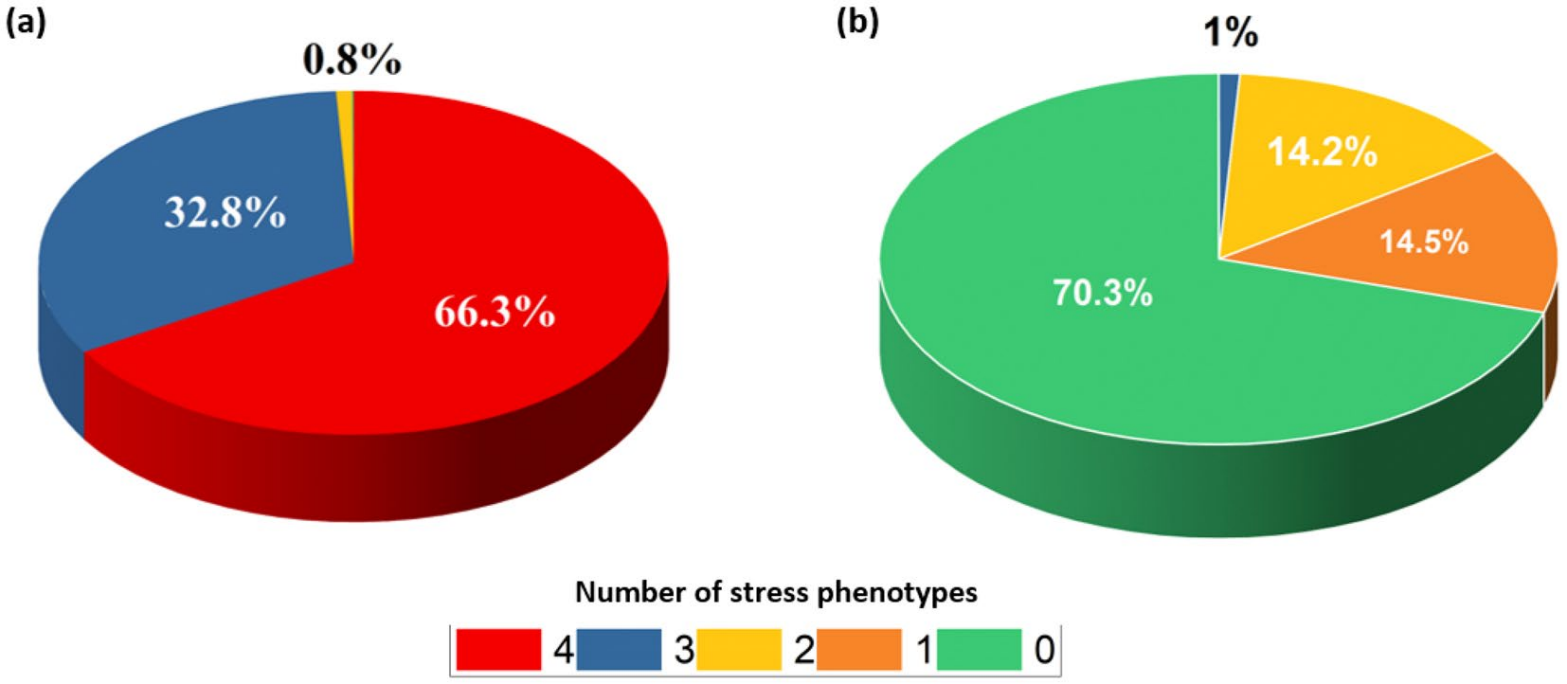
Comparison of the intervention effect on the number of individuals with different stress phenotypes within the population. (**a**) Under stress, without intervention, (**b**) Under stress, with combined intervention of botanical extracts and micronutrients. Plot representation (Red: all four stress phenotypes; Blue: any three stress phenotypes; Yellow: any two stress phenotypes; Orange: any one stress phenotype; Green: no stress phenotype).

The above population analysis includes individuals with one or multiple stress types being studied. The population is sub-grouped to categories with no stress, single stress and combination of multiple stress types. Figure 5 represents the percentage in the population with different stress phenotypes pre (Fig. 5a) and post (Fig. 5b) intervention due to the final product containing botanicals and micronutrients. It can be observed that pre intervention, the population had 66% of individuals having all four types of stress, 33% three types and about 1% with two stress types. Post intervention, the distribution changes to 70% with no stress, 14.5% with one type, 14.2% with two types and about 1% with three types of stress, indicating a substantial physiological capability of the formulation to manage stress in a population. In terms of the stress types, it is observed that in the sub-population having all stress types, the post-intervention percentage is found to be 3.01%, 14.47%, 1.35%, and 3.31% for individuals suffering from oxidative, inflammatory, physical and mental stress, respectively (See supplementary Table S3). Thus, it is noted that physical, mental and oxidative have substantial reduction in respective stress levels as compared to that for inflammation.

## Discussion

In contemporary times, stress has become integral aspect of life, leading to majority of the population experiencing its adverse effect. Modern lifestyle has resulted in various stress types which include oxidative, inflammatory, mental and physical stresses. Each of these stress types trigger several key nodes in signalling and immune pathways resulting in pathophysiological outcomes such as alleviated blood Pressure, mental stress, oxidative stress and subclinical inflammation. Further, through common nodes and feedbacks different types of stress are interconnected thereby effecting multiple stress outcomes. A systems biology modelling approach capturing the dynamics of key nodes was able to comprehend the pathophysiology of the stress types and also quantify the effect of a formulation in alleviating stress and its associated complications. The analysis was able to quantitatively demonstrate the synergistic effects of botanical extracts along with micronutrients present in Nutrilite^®^ Daily Plus, in relieving stress at multiple levels.

Botanical extracts of Gotukola, elderberry, acerola and purple carrot are in heavy demand by pharmaceutical and cosmetic industry due to their potential anti-oxidant and anti-inflammatory activity^45–48^. According to Masola et al., Gotukola exhibits anti-inflammatory activity by suppressing TNF-α production by approximately 2.5, 3.5, and tenfold at concentrations of 50, 100, and 200 μg/mL, respectively^2^. Similarly, Simonyi et al.^49^, showed the antioxidant effect of elderberry, where a 0.5-fold decrease was observed in ROS production for 400 μg/mL extract. Purple carrot’s anti-inflammatory bioactivity was investigated in an LPS-induced stress environment, revealing a 66% reduction in TNF-α levels with 13.3 µg/mL of the purple carrot fraction^18^. In addition to botanicals multivitamin supplementation also promotes physical and mental health^50^. A recent study by Pisoschi et al.^51^, has demonstrated anti-oxidant and anti-inflammatory activity of vitamins in COVID-19 therapy.

Activation of NF-κB pathway stimulates the secretion of pro-inflammatory cytokines such as TNF-α and IL-6 leading to enhanced inflammatory stress ultimately engaging the HPA axis (see Figure S5). The dose-efficacy analysis demonstrated that Vitamin-A has the maximum effect in mitigating TNF-α levels followed by acerola cherry extract and Gotukola extract indirectly through NF-κB. The individual effects for intervention for 35 days on TNF-α levels was in the order of 25% (by Vitamin-A) to 11% (by Gotukola). However, the combined formulation brought down the inflammation down by 78% as characterized by TNF-α levels in a synergistic effect lowering the NF-κB and TNF-α levels. This observation aligns with and extends the existing literature, highlighting the potential of the combined formulation to exert a more pronounced anti-inflammatory impact compared to individual components alone. The synergetic effect is compatible with the evidence reported by Patrick et al. in 2014, which shows supplementation with only Vitamin E (469 mg) reduces TNF-α levels by 59%, and with alpha-lipoic acid (ALA) (300 mg) reduces TNF-α levels by 61%. However, the combined effect of Vitamin E (469 mg) and ALA (300 mg) demonstrates a substantial 83% reduction in TNF-α levels, indicating a higher efficacy when these components are used in combination^52^. Similarly, Giasvand et al., in 2010 examine the effect of co-supplementation on TNF-α levels reported an 8% reduction with EPA (2000 mg) and an 18% reduction with Vitamin E (268 mg) individually. However, co-supplementation with EPA (2000 mg) and Vitamin E (268 mg) shows a remarkable 32% reduction in biomarker levels, emphasizing the enhanced anti-inflammatory impact achieved through their synergistic combination^53^. Interestingly, it should be noted that both the above studies use very high dosage of Vitamin E to bring about the efficacy of 61%. However, our study indicates that employing lower dosage of multiple components exerts intensified efficacy in mitigating stress levels.

Oxidative stress is directly correlated to ROS levels, which in turn interacts with PI3K/AKT, NF-κB and hypoxia-inducible factor-1α (HIF-1α), which results both in inflammatory response and heightened cortisol levels (see Figure S6). Oxidative stress is known to be influenced by high carbohydrate and calorie diets, lowered catabolic activity (sedentary lifestyle) and also enhanced consumption of processed foods^54,55^. Bumrungpert et al. in 2020, demonstrated a notable 28% reduction in oxidative stress with nutraceutical compound comprising Bittermelon fruit, Vitamin B (Biotin), Vitamin D (Cholecalciferol), Zinc, and Chromium^56^. Our investigation into a similar formulation revealed a remarkable 88% decline in ROS production, which influenced multiple nodes, thereby reducing the net oxidative stress. The data suggests potential synergistic effects as individually zinc and vitamin C showed reductions of 11%, and Acerola, Gotukola of 21% and 24%, respectively. This heightened impact can be attributed to the combined influence of vitamins E and B, alongside essential minerals, within Nutrilite^®^ Daily Plus formulation. These components appear to affect the Cortisol/SOD ratio by enhancing Superoxide Dismutase (SOD) activity and influencing cortisol levels. Acerola and Gotukola, integral to Nutrilite^®^ Daily Plus formulation, further contribute to this effect by influencing the AKT AMPK pathway, resulting in an increased antioxidant status. Moreover, Gotukola’s enhancement of the NRF anti-inflammatory pathway leads to a substantial 79% reduction in inflammation, surpassing the 18% reduction reported in the study by Bumrungpert et al^56^.

Mental stress is associated with heightened cortisol levels which is affected by different pathways resulting in activation of HPA axis. As mentioned for oxidative stress, elevated levels of ROS stimulates HIF-1α factor that stimulates secretion of cortisol via two intermediate hormones ACTH and Corticotrophin-releasing hormone (CRH)^57^. Increased levels of pro-inflammatory cytokines including TNF-α and IL-6 produced by stimulated NF-κB pathway, are also instrumental in evoking mental stress by upregulating cortisol levels. Severity of mental stress in response to increased cortisol levels is reflected in a high PSS (Figure S7). Administration of botanicals, minerals and vitamins diminish TNF-α and IL-6 levels thereby reducing cortisol levels. Simulations demonstrated that acerola was the most effective with reduction by 22%, followed by combination of Vitamin A + C by 16%. However, Nutrilite^®^ Daily Plus formulation brought down the mental stress scores by 72% demonstrating the synergistic effect by effecting multiple nodes (see Figure S7).

All types of stress mentioned above, including inflammatory, oxidative and mental stress, can manifest as physical stress, as evidenced by increased heart rate and blood pressure. Renin-angiotensin system (RAS) is involved in governing blood pressure^58^. In certain anomalies, activated RAS releases excessive angiotensin II (Ang II) that results in hyper secretion of aldosterone and subsequently increased blood pressure. TNF-α functions as a crucial factor in development of RAS dependent hypertension as inhibition of TNF-α levels abrogated increased blood pressure^59^. As per the model, multiple components of the formulation including Gotukola, acerola, vitamin A, vitamin C, zinc, selenium, magnesium lower the levels of pro-inflammatory cytokines thus attenuating RAS activation and reduce elevated blood pressure. Simulation of Nutrilite^®^ Daily Plus formulation, composed of these botanicals and micronutrients reduces BP levels by 79%. In recent studies by Majeed et al. (2023) and Salve et al. (2019), the adaptogenic Ayurvedic herb Ashwagandha has been investigated for its stress-reducing properties, with an additional focus on its potential to improve sleep quality^60,61^. The research, reporting a decrease in the PSS score ranging between 33 and 45% across varied Ashwagandha doses, highlights its efficacy in stress management. In a parallel exploration by Gasparini et al. (2016), the stress-reducing properties of a combination of herbs, including valerian, hops, passion flower, ginseng, and rhodiola, were examined in separate groups. The overall reduction in stress levels ranged from 23 and 35%, emphasizing the potential need for additional stress-relieving compounds^62^.

A comprehensive understanding of stress modulation by the stress relieving compounds or botanicals involve recognizing the role of the ACTH pathway in reducing cortisol levels, a key correlate of mental stress. The botanicals such as Acerola, Gotukola, Elderberry present in our formulation also acts on NF-κB pathway showing a reduction in IL-6, TNF-α levels which further reduces the cortisol levels. Individually, these botanicals demonstrated reductions of around 22%, 24%, and 11%, respectively. Additionally, the inclusion of Vitamin A and the combination of Vitamin A + C showed substantial drops of 26%, 17%, and 19%, underscoring the significant impact of vitamins and minerals on stress reduction. By integrating these potent botanicals, vitamins, and minerals, our formulation strategically targets multiple pathways, resulting in a remarkable 72% reduction in mental stress scores, directly associated with cortisol levels. This comprehensive approach signifies the potential of our formulation to offer effective stress relief, addressing the intricate interplay of various stress-related pathways.

Structuring various pathways and nodes that contribute towards development of four types of stress within a unified network provides a dynamic mechanistic insight of interconnected network to design formulation that can ameliorate stress related complications. Simulation of a mathematical model incorporating the comprehensive network with botanicals, vitamins and minerals renders a formulation, Nutrilite^®^ Daily Plus, that may possess potential stress relieving property at both individual and population levels, by significantly attenuating activation of NF-κB pathway, TNF-α, ROS, cortisol (PSS) and SBP levels. The study demonstrated that the Nutrilite^®^ Daily Plus containing several botanicals and micronutrients which potentially affects multiple nodes in the stress pathway demonstrates a broad efficacy among individual in a population with varied stress types and with varied stress levels. The results from thee in-silico analysis presented here could form a basis for a clinical study to demonstrate its effectiveness. Furthermore, a systems biology modelling approach could provide a design principle in developing formulations using botanical extracts, nutraceuticals and micronutrients in the overall wellness.

## Conclusion

The predictive mathematical model dedicated to understanding inflammation offers a profound perspective on the complex interconnections among various stress types. These stress types encompass oxidative stress, which is related to damage caused by free radicals; inflammatory stress, which pertains to the body’s response to harmful stimuli; physical stress, stemming from bodily wear and tear or injury; and mental stress, which relates to psychological pressures and challenges. By using this mathematical model, we have simulated the behaviour of specific botanicals and micronutrients. These elements are pivotal constituents of the Nutrilite^®^ Daily Plus formulation. The simulation study revealed noteworthy shifts in an array of stress-associated parameters. Such modifications induced by the botanicals and micronutrients aren’t merely statistical or theoretical changes. They hold significant practical implications. The alterations suggest a potential reduction in the complications associated with prolonged or chronic stress. Over time, minimizing these complications could lead to a heightened overall quality of life. This could mean fewer health issues related to stress, better mental well-being, and a general improvement in day-to-day living for individuals while addressing the micronutritional gap using the Nutrilite^®^ Daily Plus formulation.

## Methods

### Model description

The study employs mathematical modelling and simulations using a customized version of Metflux’s proprietary subclinical inflammation model. The model is formulated using ordinary differential equations depicting the signalling pathways which were solved in MATLAB using ordinary differential equation (ODE) solver Ode15s (File S1, Fig. 1). The model includes various signalling pathways and species such as inflammasome signalling, NF-κB, NRF2-KEAP1, PI3K-AKT-mTOR, JAK1-STAT3 & MAPK, IL-6, IL-10 and TNF-α signaling, insulin and glucagon metabolism, LPS-induced inflammation, renin-angiotensin signaling, etc. To define a stressed environment, the model variables cortisol, SOD, pIRS/IRS ratio, pAKT/AKT ratio, TNF-α, and ROS production percentage are benchmarked against available literature data. In the mathematical model, stress is induced by perturbing molecular mechanisms, particularly by modifying insulin signalling through AKT, PI3K pathways and inflammatory signalling pathway involving IL-6 and NF-κB pathway. During insulin stimulation, AKT-induced phosphorylation of FOXO1 results in its cytosolic accumulation that triggers LPS activation. Subsequently, the expression of IL-6 and TNF-α, which are indicative of changes in pressure overload, disrupts the balance of STAT3 and IL-10. This cascade of events ultimately modulates LPS, NF-κB pathway and HPA axis notably altering angiotensin and cortisol levels. Similarly, the oxidative stress in the model is attained by disturbing the interplay between antioxidant enzymes such as SOD and non-enzymatic moieties like GSH and the NRF2 pathway. This leads to excessive ROS generation, a key determinant of enhanced oxidative stress. Thus, in summary, the model predominantly involves NF-κB and NRF2 pathways, effectively capturing the transcription factors responsible for stress-related signalling (Fig. 1).

The model’s stress input influences various output parameters such as ROS production, TNF-α, BP and cortisol levels. These output parameters are associated with oxidative, inflammatory, physical, and mental stress, respectively. The effect of intervention input is therefore studied on these model output parameters to determine oxidative, inflammatory, physical, and mental stress (Figure S8).

### Intervention description

The active ingredients in the Nutrilite^®^ Daily Plus formulation are demonstrated to have antioxidant and anti-inflammatory effects over diverse stress parameters. The principle components of Nutrilite^®^ Daily Plus are botanicals including Gotukola (*Centella asciatica* extract) with 15% triterpenoids, acerola cherry, elderberry and purple carrot, vitamins including vitamin A, D, E, K, C, B vitamins, and minerals such as iodine, copper, manganese, molybdenum, selenium, zinc, chromium, iron, magnesium, calcium and phosphorus (Table S4 and S5).

### Output parameters

Exogenous as well as endogenous stressors can affect physical and mental well-being by modulating oxidative and inflammatory pathways. Usually, the stressful condition is diagnosed by elevated levels of systolic blood pressure (SBP) and diastolic blood pressure (DBP) beyond 120 mm Hg and 80 mm Hg respectively. This is initiated with excessive release of stress-hormone cortisol, triggered by stimulated HPA axis. Individuals with cortisol levels falling within the range of 140–690 nmol/L are considered to be psychologically healthy whereas elevation in these levels are associated with mental stress that is diagnosed by measuring PSS. The PSS score is obtained by summing the points awarded to the ten items which ranges from 0 to 40, where: 0–13– > Low stress; 14–26– > Moderate stress; and 27–40– > High perceived stress^63^. Elevated levels of cortisol influence oxidative stress pathways by increasing ROS production. Overproduction of ROS serves as a key indicator of stressful condition. Under stressed condition the levels of ROS increase more than 10% compared to healthy state, resulting in suppression of beneficial anti-oxidant enzymes including catalase, GSH below normal levels and increase in MDA levels^64^. Increased oxidative stress disrupts activation of inflammatory pathways, thereby stimulating excessive release of various cytokines from activated immune cells. Inflammatory stress is attained, when the serum levels of TNF-α (0–1.5 pg/mL) surpass the normal levels^65^. The deviation from healthy range value was considered as stressed condition.

### Model validation

The credibility and integrity of subclinical inflammation model is assessed using literature based data. Multiple research articles are retrieved that contain the information about the effect of supplementation with botanicals and micronutrients, including vitamins and minerals (Table S1 and S4), on various parameters of oxidative, inflammatory, physical and mental stress. The extracted data is used to validate the model as well as for independent analysis.

### In-silico population analysis

An in-silico population of 1000 adult males and females (20–80 years) with varying stress levels (ROS, TNF-α, SBP and PSS score) was generated. The individual and combined effects of botanical extracts, micronutrients were evaluated by simulating the validated model with in silico population suffering from oxidative, inflammatory, physical and mental stress.

### Supplementary Information

Supplementary Information.

## Data Availability

The datasets used and/or analysed during the current study available from the corresponding author on reasonable request.

## References

[1] Camfield DA (2013). The effects of multivitamin supplementation on diurnal cortisol secretion and perceived stress. Nutrients.

[2] Masola B, Oguntibeju OO, Oyenihi AB (2018). *Centella asiatica* ameliorates diabetes-induced stress in rat tissues via influences on antioxidants and inflammatory cytokines. Biomed. Pharmacother..

[3] Potashnik R, Bloch-Damti A, Bashan N, Rudich A (2003). IRS1 degradation and increased serine phosphorylation cannot predict the degree of metabolic insulin resistance induced by oxidative stress. Diabetologia.

[4] Yung HW, Charnock-Jones DS, Burton GJ (2011). Regulation of AKT phosphorylation at Ser473 and Thr308 by endoplasmic reticulum stress modulates substrate specificity in a severity dependent manner. PLoS One.

[5] Choi M-J (2016). Protective effects of *Centella asiatica* leaf extract on dimethylnitrosamine-induced liver injury in rats. Mol. Med. Rep..

[6] Kumari S, Deori M, Elancheran R, Kotoky J, Devi R (2016). In vitro and In vivo antioxidant, anti-hyperlipidemic properties and chemical characterization of *Centella asiatica* (L.) Extract. Front. Pharmacol..

[7] Zagórska-Dziok M, Ziemlewska A, Bujak T, Nizioł-Łukaszewska Z, Hordyjewicz-Baran Z (2021). Cosmetic and dermatological properties of selected ayurvedic plant extracts. Molecules.

[8] Gray NE (2017). *Centella asiatica* attenuates mitochondrial dysfunction and oxidative stress in Aβ-exposed hippocampal neurons. Oxid. Med. Cell. Longev..

[9] Jagadeesan S (2019). *Centella asiatica* prevents chronic unpredictable mild stress-induced behavioral changes in rats. Biomed. Res. Ther..

[10] Park JH (2017). Anti-inflammatory effect of titrated extract of *Centella asiatica* in phthalic anhydride-induced allergic dermatitis animal model. Int. J. Mol. Sci..

[11] Sari DCR (2019). *Centella asiatica* (Gotu kola) ethanol extract up-regulates hippocampal brain-derived neurotrophic factor (BDNF), tyrosine kinase B (TrkB) and extracellular signal-regulated protein kinase 1/2 (ERK1/2) signaling in chronic electrical stress model in rats. Iran. J. Basic Med. Sci..

[12] Shinomol GK, Muralidhara (2008). Effect of *Centella asiatica* leaf powder on oxidative markers in brain regions of prepubertal mice in vivo and its in vitro efficacy to ameliorate 3-NPA-induced oxidative stress in mitochondria. Phytomedicine.

[13] Dias FM (2014). Acerola (*Malpighia emarginata* DC.) juice intake protects against alterations to proteins involved in inflammatory and lipolysis pathways in the adipose tissue of obese mice fed a cafeteria diet. Lipids Health Dis..

[14] Souza NC (2020). Anti-inflammatory and antixidant properties of blend formulated with compounds of *Malpighia emarginata* D.C (acerola) and *Camellia sinensis* L. (green tea) in lipopolysaccharide-stimulated RAW 264.7 macrophages. Biomed. Pharmacother..

[15] Olejnik A (2016). A gastrointestinally digested *Ribes nigrum* L. fruit extract inhibits inflammatory response in a co-culture model of intestinal Caco-2 cells and RAW264.7 macrophages. J. Agric. Food Chem..

[16] Frøkiær H (2012). Astragalus root and elderberry fruit extracts enhance the IFN-β stimulatory effects of *Lactobacillus acidophilus* in murine-derived dendritic cells. PLoS One.

[17] Tiralongo E, Wee SS, Lea RA (2016). Elderberry supplementation reduces cold duration and symptoms in air-travellers: A randomized, double-blind placebo-controlled clinical trial. Nutrients.

[18] Metzger BT, Barnes DM, Reed JD (2008). Purple carrot (*Daucus carota* L.) polyacetylenes decrease lipopolysaccharide-induced expression of inflammatory proteins in macrophage and endothelial cells. J. Agric. Food Chem..

[19] Zhang H (2017). Bioaccessibility, bioavailability, and anti-inflammatory effects of anthocyanins from purple root vegetables using mono- and co-culture cell models. Mol. Nutr. Food Res..

[20] Soares GR (2018). Protective effects of purple carrot extract (*Daucus carota*) against rat tongue carcinogenesis induced by 4-nitroquinoline 1-oxide. Med. Oncol..

[21] Petiz LL (2017). Role of vitamin A oral supplementation on oxidative stress and inflammatory response in the liver of trained rats. Appl. Physiol. Nutr. Metab..

[22] Ohsaki Y (2006). Vitamin K suppresses lipopolysaccharide-induced inflammation in the rat. Biosci. Biotechnol. Biochem..

[23] Haddad Kashani H (2018). The effects of vitamin D supplementation on signaling pathway of inflammation and oxidative stress in diabetic hemodialysis: A randomized, double-blind, placebo-controlled trial. Front. Pharmacol..

[24] Roberts LJ (2007). The relationship between dose of vitamin E and suppression of oxidative stress in humans. Free Radic. Biol. Med..

[25] Abdoulhossein D (2018). Effect of vitamin C and vitamin E on lung contusion: A randomized clinical trial study. Ann. Med. Surg..

[26] Mazloom Z, Ekramzadeh M, Hejazi N (2013). Efficacy of supplementary vitamins C and E on anxiety, depression and stress in type 2 diabetic patients: A randomized, single-blind, placebo-controlled trial. Pak. J. Biol. Sci. PJBS.

[27] Zaringhalam J (2016). Long-term treatment by vitamin B(1) and reduction of serum proinflammatory cytokines, hyperalgesia, and paw edema in adjuvant-induced arthritis. Basic Clin. Neurosci..

[28] Lappas M, Permezel M (2011). The anti-inflammatory and antioxidative effects of nicotinamide, a vitamin B(3) derivative, are elicited by FoxO_3_ in human gestational tissues: Implications for preterm birth. J. Nutr. Biochem..

[29] Mazur-Bialy AI, Pocheć E (2016). Riboflavin reduces pro-inflammatory activation of adipocyte-macrophage co-culture. Potential application of vitamin B2 enrichment for attenuation of insulin resistance and metabolic syndrome development. Molecules.

[30] Ganji SH, Qin S, Zhang L, Kamanna VS, Kashyap ML (2009). Niacin inhibits vascular oxidative stress, redox-sensitive genes, and monocyte adhesion to human aortic endothelial cells. Atherosclerosis.

[31] He W (2018). Vitamin B5 reduces bacterial growth via regulating innate immunity and adaptive immunity in mice infected with *Mycobacterium tuberculosis*. Front. Immunol..

[32] Huang S-C, Wei JC-C, Wu DJ, Huang Y-C (2010). Vitamin B(6) supplementation improves pro-inflammatory responses in patients with rheumatoid arthritis. Eur. J. Clin. Nutr..

[33] Ebrahimi E, Khayati Motlagh S, Nemati S, Tavakoli Z (2012). Effects of magnesium and vitamin b6 on the severity of premenstrual syndrome symptoms. J. Caring Sci..

[34] Agrawal S, Agrawal A, Said HM (2016). Biotin deficiency enhances the inflammatory response of human dendritic cells. Am. J. Physiol. Cell Physiol..

[35] Asbaghi O (2021). Effects of folic acid supplementation on inflammatory markers: A grade-assessed systematic review and dose-response meta-analysis of randomized controlled trials. Nutrients.

[36] Zadeh-Ardabili PM, Rad SK, Rad SK, Movafagh A (2019). Antidepressant-like effects of fish, krill oils and Vit B12 against exposure to stress environment in mice models: Current status and pilot study. Sci. Rep..

[37] Chellappa AR, Karunanidhi S (2012). Supplementation with iron and zinc selectively improves cognitive and behavioral functions in female adolescents. Int. J. Chem. Eng. Appl..

[38] Bao B (2010). Zinc decreases C-reactive protein, lipid peroxidation, and inflammatory cytokines in elderly subjects: A potential implication of zinc as an atheroprotective agent. Am. J. Clin. Nutr..

[39] Zemel MB, Sun X (2008). Dietary calcium and dairy products modulate oxidative and inflammatory stress in mice and humans. J. Nutr..

[40] Soriguer F (2011). Iodine intakes of 100–300 μg/d do not modify thyroid function and have modest anti-inflammatory effects. Br. J. Nutr..

[41] Kim Y (2014). Antioxidant and anti-inflammatory effects of selenium in oral buccal mucosa and small intestinal mucosa during intestinal ischemia-reperfusion injury. J. Inflamm..

[42] Owumi SE, Dim UJ (2019). Manganese suppresses oxidative stress, inflammation and caspase-3 activation in rats exposed to chlorpyrifos. Toxicol. Rep..

[43] Dmitrašinović G (2016). ACTH, cortisol and IL-6 levels in athletes following magnesium supplementation. J. Med. Biochem..

[44] Ward JD, Spears JW (1999). The effects of low-copper diets with or without supplemental molybdenum on specific immune responses of stressed cattle. J. Anim. Sci..

[45] Kunjumon R, Johnson AJ, Baby S (2022). *Centella asiatica*: Secondary metabolites, biological activities and biomass sources. Phytomed. Plus.

[46] Ferreira SS, Martins-Gomes C, Nunes FM, Silva AM (2022). Elderberry (*Sambucus nigra* L.) extracts promote anti-inflammatory and cellular antioxidant activity. Food Chem. X.

[47] Olędzki R, Harasym J (2024). Acerola (*Malpighia emarginata*) anti-inflammatory activity: A review. Int. J. Mol. Sci..

[48] Rasheed H (2022). Delving into the nutraceutical benefits of purple carrot against metabolic syndrome and cancer: A review. Appl. Sci..

[49] Simonyi A (2015). Inhibition of microglial activation by elderberry extracts and its phenolic components. Life Sci..

[50] Oliver-Baxter JM, Whitford HS, Turnbull DA, Bond MJ (2018). Effects of vitamin supplementation on inflammatory markers and psychological wellbeing among distressed women: A randomized controlled trial. J. Integr. Med..

[51] Pisoschi AM (2022). Antioxidant, anti-inflammatory and immunomodulatory roles of vitamins in COVID-19 therapy. Eur. J. Med. Chem..

[52] Basu P, Shah N, Aloysius M, Junior R (2014). Effect of vitamin E and alpha lipoic acid in nonalcoholic fatty liver disease: A randomized, placebo-controlled, open-label, prospective clinical trial (VAIN trial). Open J. Gastroenterol..

[53] Ghiasvand R (2010). Effect of eicosapentaenoic acid (EPA) and vitamin e on the blood levels of inflammatory markers, antioxidant enzymes, and lipid peroxidation in Iranian basketball players. Iran. J. Public Health.

[54] Jiang S, Liu H, Li C (2021). Dietary regulation of oxidative stress in chronic metabolic diseases. Foods.

[55] Martínez Leo EE, Peñafiel AM, Hernández Escalante VM, Cabrera Araujo ZM (2021). Ultra-processed diet, systemic oxidative stress, and breach of immunologic tolerance. Nutrition.

[56] Bumrungpert A, Pavadhgul P, Chongsuwat R, Komindr S (2020). Nutraceutical improves glycemic control, insulin sensitivity, and oxidative stress in hyperglycemic subjects: A randomized, double-blind, placebo-controlled clinical trial. Nat. Prod. Commun..

[57] Harrell CS, Rowson SA, Neigh GN (2015). Pharmacological stimulation of hypoxia inducible factor-1α facilitates the corticosterone response to a mild acute stressor. Neurosci. Lett..

[58] Yim HE, Yoo KH (2008). Renin-angiotensin system—considerations for hypertension and kidney. Electrolyte Blood Press.

[59] Satou R, Penrose H, Navar LG (2018). Inflammation as a regulator of the renin-angiotensin system and blood pressure. Curr. Hypertens. Rep..

[60] Majeed M, Nagabhushanam K, Mundkur L (2023). A standardized Ashwagandha root extract alleviates stress, anxiety, and improves quality of life in healthy adults by modulating stress hormones: Results from a randomized, double-blind placebo-controlled study. Medicine.

[61] Salve J, Pate S, Debnath K, Langade D (2019). Adaptogenic and anxiolytic effects of ashwagandha root extract in healthy adults: A double-blind, randomized, placebo-controlled clinical study. Cureus.

[62] Gasparini M, Aurilia C, Lubian D, Testa M (2016). Herbal remedies and the self-treatment of stress: An Italian survey. Eur. J. Integr. Med..

[63] Cohen S, Kamarck T, Mermelstein R (1983). A global measure of perceived stress. J. Health Soc. Behav..

[64] More GK, Makola RT (2020). In-vitro analysis of free radical scavenging activities and suppression of LPS-induced ROS production in macrophage cells by *Solanum sisymbriifolium* extracts. Sci. Rep..

[65] Kondkar AA (2018). Elevated levels of plasma tumor necrosis factor alpha in patients with pseudoexfoliation glaucoma. Clin. Ophthalmol..

