# Supplementary material for: A systems biology-based mathematical model demonstrates the potential anti-stress effectiveness of a multi-nutrient botanical formulation
Source: Sci Rep. 2024 Apr 26;14:9582. doi: 10.1038/s41598-024-60112-8 (PMC11053000; doi:10.1038/s41598-024-60112-8)
Supplement: Supplementary file 1 — Supplementary Information. [file 41598_2024_60112_MOESM1_ESM.docx]

**A systems biology-based mathematical model demonstrates the potential anti-stress effectiveness of a multi-nutrient botanical formulation**

**Abha Saxena^1^, Kaushiki S. Prabhudesai^1^, Aparna Damle^2^, Shyam Ramakrishnan^3^, Palaniyamma Durairaj^2^, Sumathi Kalankariyan^1^, AB Vijayalakshmi^1^ , KV Venkatesh^1,4,*^**

^1^MetFlux Research Private Limited, Mumbai, Maharashtra, India

^2^Amway Global Services India Pvt. Ltd.

^3^Amway Corporation, USA

^4^Department of Chemical Engineering, Indian Institute of Technology Bombay, Mumbai, Maharashtra, India

* Corresponding Author (KVV)

E-mail: venks@metflux.in

[venks@iitb.ac.in](mailto:venks@iitb.ac.in)

**Supplementary File**

**Model validation**

In order to mimic healthy and stressed conditions, some key parameters including cortisol, SOD, pIRS/IRS ratio, pAKT/AKT ratio, TNF-α, and percent ROS production are determined by referring the published literature data (Figure S1). The fold changes observed in literature data under stress condition for the model variables cortisol^1^, SOD^2^, pIRS/IRS ratio^3^, pAKT/AKT ratio^4^, TNF-α^5^, and ROS production percentage^6^ are 1.79 ± 0.065, 0.44 ± 0.025, 0.55 ± 0.1, 0.45 ± 0.005, 1.56 ± 0.107, 3.25 ± 0.5, respectively. The model has been benchmarked such that the fold change dynamics are similar to the available literature for the stressed environment. The fold change values observed in the model are 1.53 ± 0.28, 0.5 ± 0.0005, 0.44 ± 0.0005, 0.55 ± 0.0005, 1.63 ± 0.625, 2.92 ± 0.575, for cortisol, SOD, pIRS/IRS ratio, pAKT/AKT ratio, TNF-α, and ROS production percentage, respectively.
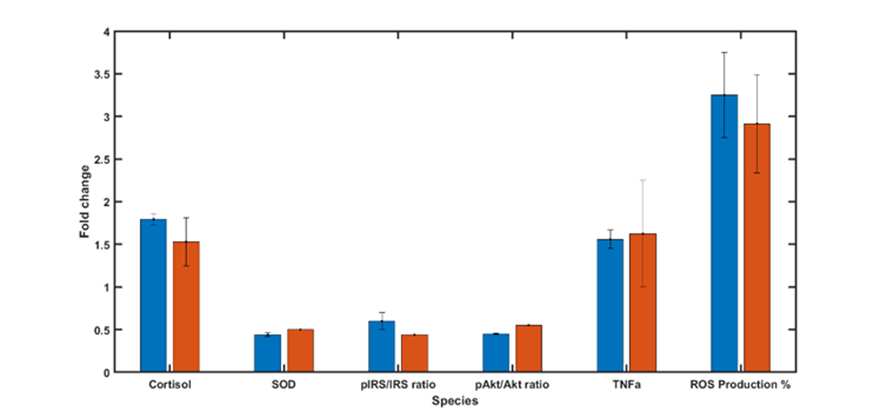


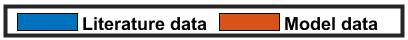


**Figure S1: Model validation for different model variables with respect to literature data.** (a) Cortisol, (b) SOD, (c) pIRS/IRS ratio, (d) pAKT/AKT ratio, (e) TNF-α, and (f) ROS production percentage. Plot representation (Blue: Literature data; Red: Model data).

The reliability and effectiveness of this subclinical inflammatory model is further evaluated by comparing the results published in literature. Fold change values of TNF-α, SBP, ROS and IL-6 obtained after simulating the model with literature based intervention data, were observed to align with the published literature (Figure S2a-d, Table S1).

It is noteworthy that, similar to individual components, combinations of minerals and vitamins also exhibit similar fold change when simulated in model. With respect to ROS, co-supplementation of vitamin D and iron in model exhibit fold change of 0.99 which closely resembles the results of literature-based analysis (fold change: 0.85) (Figure S2c). Similarly, when vitamin C and zinc are administered together, fold reduction of 0.87 and 0.89 is observed in literature and model respectively (Figure S2c). Additionally, vitamin C and vitamin A together reduce IL-6 levels similar to individual components, with fold change of 0.51 and 0.81 as seen in literature and model respectively (Figure S2d). The results suggest that model is capable of accurately estimating the combinatorial effect of micronutrients.


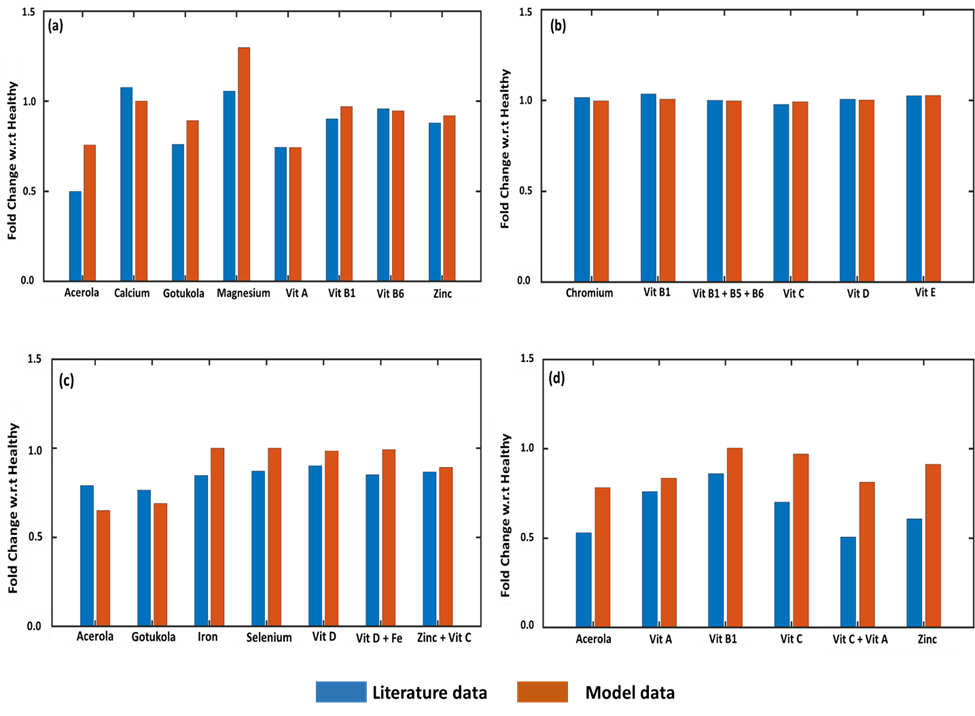


**Figure S2: Effect of botanicals and micronutrients on the levels of a) TNF-α b) SBP, c) ROS and d) IL6 as per literature and subclinical inflammatory model.** Each bar graph represents the fold change values observed in literature and model-based analysis in stressed conditions when supplemented with corresponding botanicals or micronutrients independently as well as in combination with respect to healthy state.

**Table S1: Fold change values of various stress-related parameters post intervention as shown in literature.**

| **Stress-markers** | **Intervention components** | **Fold change** | **References** |
| --- | --- | --- | --- |
| TNF-α | Vitamin B1 | 0.90 | Gonza´lez-Ortiz et al., 2011^7^ |
|  | Vitamin A | 0.74 | Boyali et al., 2016^8^ |
|  | Vitamin B6 | 0.96 | Huang et al., 2010^9^ |
|  | Magnesium | 1.06 | Mousavi et al., 2021^10^ |
|  | Calcium | 1.08 | Yang et al., 2015^11^ |
|  | Zinc | 0.88 | Mekswan et al., 2014^12^ |
|  | Gotukola | 0.76 | Kumari et al.,2016^13^, Intararuchikul 2019^14^, Masola et al.,2018^15^, Giribabu et al., 2020^16^, Choi et al.,2016^17^, |
|  | Acerola | 0.50 | Hu et al., 2020^18^ |
| SBP | Vitamin B1 | 1.03 | Al-Attas et al.,2013^18^ |
|  | Vitamin D | 1.01 | Yiu et al., 2013^18^ |
|  | Vitamin C | 0.98 | de Oliveira2015^19^ |
|  | Vitamin E | 1.03 | takahashi2013^20^ |
|  | Chromium | 1.02 | nussbaumerova2017^21^ |
|  | Vitamin B1 + B5 + B6 | 1.00 | yanni2019^22^ |
| ROS | Vitamin D | 0.90 | Foroozanfard et al.,2015^23^ |
|  | Iron | 0.85 | Khoshfetrat et al., 2013^24^ |
|  | Selenium | 0.87 | Farrokhian et al.,2016^25^ |
|  | Gotu kola | 0.69 | Mairuae et al., 2019^26^ |
|  | Acerola | 0.65 | Alvarez-Suarez et al., 2017^27^ |
|  | Vitamin D + Iron | 0.85 | Abiri et al., 2020^28^ |
|  | Vitamin C+ Zinc | 0.87 | Saad-hussein et al., 2019^29^ |
| IL-6 | Vitamin B1 | 0.86 | yanni2019^22^ |
|  | Vitamin A | 0.76 | Ramezani et al., 2014^30^,  Cai_2019^31^ |
|  | Vitamin C | 0.70 | Cai_2019^31^ |
|  | Zinc | 0.61 | de_moura et al., 2020^32^ |
|  | Acerola | 0.53 | Hu et al., 2020^18^ |
|  | Vitamin C + Vitamin A | 0.51 | Cai_2019^31^ |

## **Steady-state dynamics**

The model is customized to deduce sensitive parameters when perturbed to simulate various stress conditions. The induced stress causes the variation in ROS production, cortisol, and TNF-α levels (Figure S3). The elevated steady-state levels of clinical parameters related to different stress types can be observed when induced with stress compared to healthy individuals.

Figure S3a shows that the ROS production levels peak at 5% in the healthy scenario but are upregulated by approximately 37-fold, increasing the production of ROS species to 172% during stress. Cortisol levels are also elevated by 21 times due to stress, increasing the peak value to 10^6^ ng/mL, compared to the healthy peak value of 5 ng/mL (Figure S3b). Similarly, a 57-fold increase in stress is associated with increasing TNF-α levels with a peak value of 2.2x10^−3^ pg/mL, compared to the average baseline value of 3.81x10^−5^ pg/mL (Figure S3c).


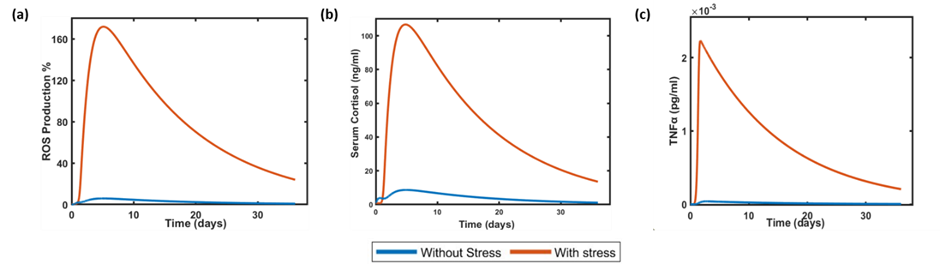


**Figure S3: Steady-state dynamics for stress induction on clinical stress parameters.** (a) ROS production, (b) Cortisol levels, and (c) TNF- α levels. Plot representation (Blue: Healthy condition, without stress; Red: Under stress).

**Table S2:** **Statistical analysis comparing the intervention effect on the clinical stress parameters.**

| **Stress parameters ^** | **Baseline healthy** | **Without intervention *** | **p-value (1-2) *** | **Only with micronutrients*** | **p-value (1-3) *** | **Only with botanical extract*** | **p-value (1-4) *** | **With combination of botanical extracts and micronutrients*** |
| --- | --- | --- | --- | --- | --- | --- | --- | --- |
| Oxidative  (ROS production %) | <10 | 248.42 ± 62.98 | 3.7 x 10^-4^ | 149.93 ± 27.77 | 7.3 x 10^-6^ | 99.77 ± 28.11 | 5.5 x 10^-7^ | 29.92 ± 11.25 |
| Inflammatory  (TNF-α - x10^−3^ pg/ml) | 0-1.5 | 38 ± 5.2 | 5.7 x 10^-7^ | 11 ± 3.2 | 3 x 10^-6^ | 18 ± 3.4 | 2.6 x 10^-7^ | 8 ± 2.9 |
| Physical  (BP - mm of Hg) | 100-120 | 160.71 ± 7.92 | 3.4 x 10^-7^ | 119.88 ± 4.96 | 9.4 x 10^-6^ | 129.91 ± 5.16 | 1.9 x 10^-7^ | 115.41 ± 4.33 |
| Mental  (PSS) | 0 | 32.72 ± 8.45 | 5.9 X 10^-4^ | 22.39 ± 6.60 | 1.8 x 10^-4^ | 15.52 ± 4.02 | 7.5 x 10^-6^ | 9.04 ± 3.38 |

* Mean ± Standard deviation.

(1-2)* - statistical significance between Without Nutrilite® Daily plus and only with micronutrients

(1-3)* - statistical significance between Without Nutrilite® Daily plus and only with Gotukola mixture

(1-4)* - statistical significance between Without Nutrilite® Daily plus and only with Daily plus

^


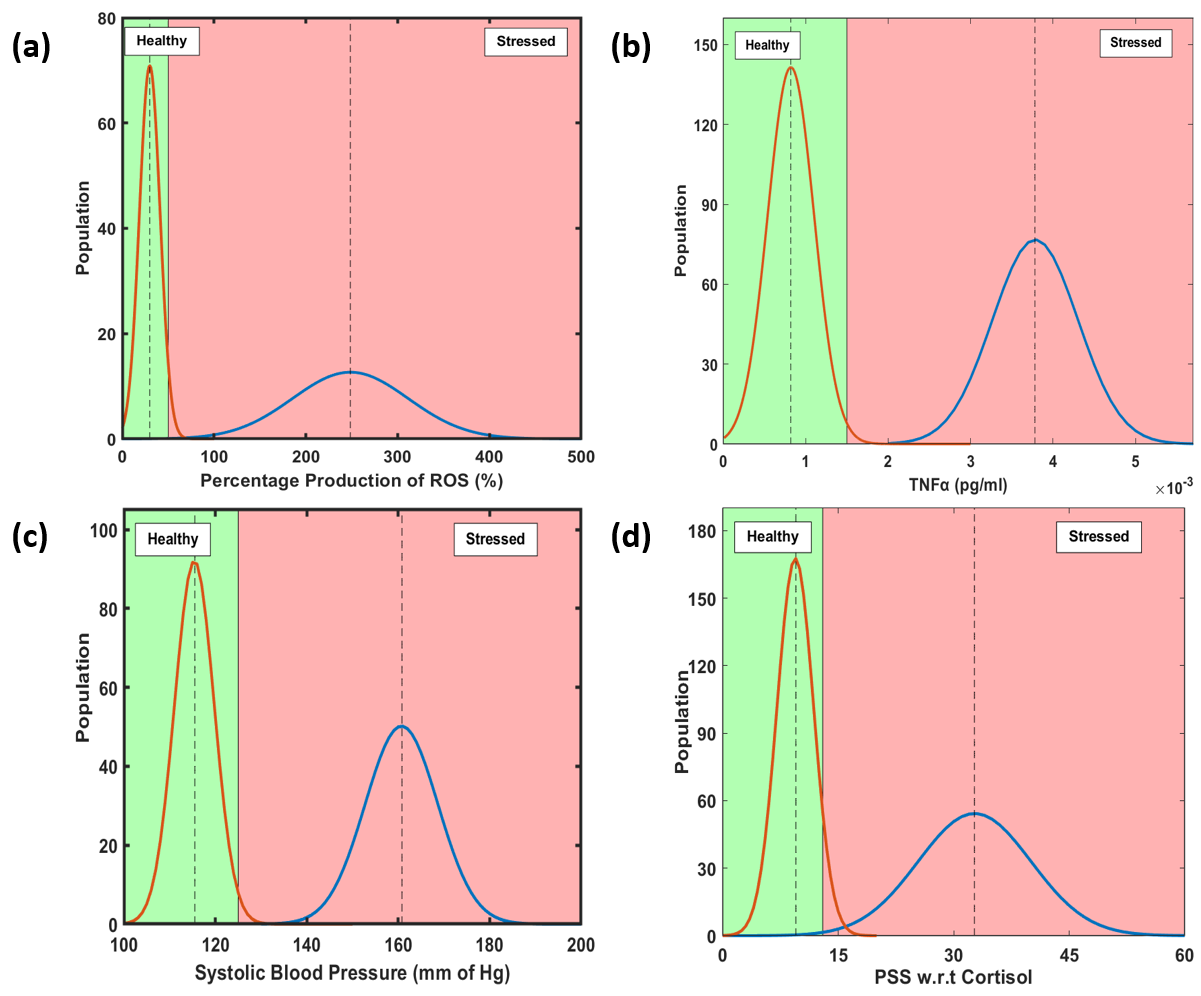


**Figure S4: Population distribution analysis comparing intervention effect on clinical stress parameters for respective stress types.** (a) ROS production for Oxidative stress, (b) TNF-α levels for Inflammatory stress, (c) BP levels for Physical stress, and (d) PSS score for Mental stress. Plot representation (Blue line: Under stress, without intervention; Red line: Under stress, with combined intervention of botanical extracts and micronutrients, Green area: Healthy condition, Red area: Stressed condition).

**Table S3: Statistical analysis comparing the intervention effect on the number of individuals with different stress phenotypes within the population**

| **Number of stress phenotypes** | **Stressed Population** | | **Stress Type** | **Stressed Population (%)** | | **Stressed Population (number of individuals)** | |
| --- | --- | --- | --- | --- | --- | --- | --- |
|  | **Percentage (%)** | **Number of individuals** |  | **Without intervention** | **With intervention** | **Without intervention** | **With intervention** |
| 4 | 66.3 | 663 | Oxidative stress | 100 | 3.01 | 663 | 20 |
|  |  |  | Inflammatory stress | 100 | 14.4 | 663 | 95 |
|  |  |  | Physical stress | 100 | 1.35 | 663 | 9 |
|  |  |  | Mental stress | 100 | 3.31 | 663 | 22 |
| 3 | 32.8 | 328 | Oxidative stress | 97.2 | 0.3 | 319 | 1 |
|  |  |  | Inflammatory stress | 96.03 | 12.5 | 315 | 41 |
|  |  |  | Physical stress | 100 | 1.22 | 328 | 4 |
|  |  |  | Mental stress | 90.2 | 5.18 | 296 | 17 |
| 2 | 0.08 | 8 | Oxidative stress | 87.5 | 0 | 7 | 0 |
|  |  |  | Inflammatory stress | 100 | 12.5 | 8 | 1 |
|  |  |  | Physical stress | 12.5 | 0 | 1 | 0 |
|  |  |  | Mental stress | 12.5 | 12.5 | 1 | 1 |
| 1 | 0.01 | 1 | Oxidative stress | 0 | 0 | 0 | 0 |
|  |  |  | Inflammatory stress | 0 | 0 | 0 | 0 |
|  |  |  | Physical stress | 100 | 0 | 1 | 0 |
|  |  |  | Mental stress | 0 | 0 | 0 | 0 |


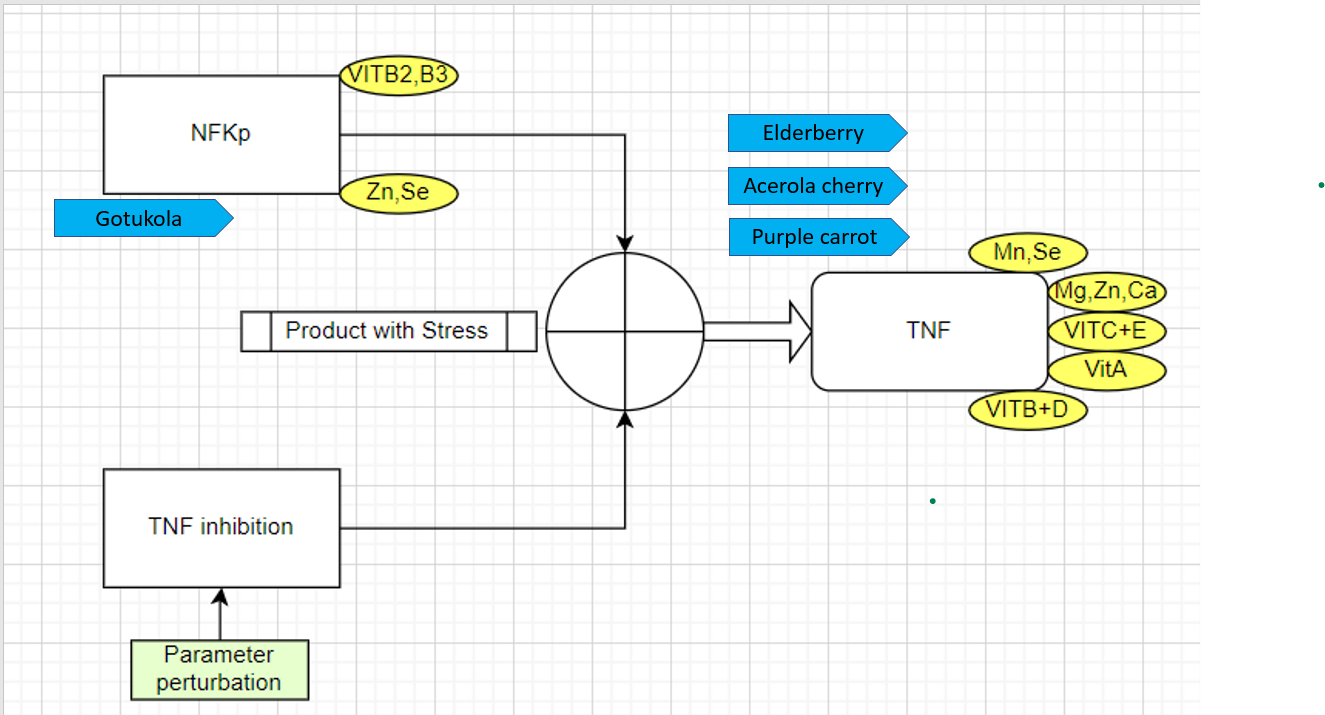


**Figure S5: Synergistic Modulation of Stress-Induced TNF Pathway and NFKB Pathways by TNF Inhibition Factor and Micronutrients-Gotukola Mixture for Effective Stress Management.**

**Effective pathways:** The stress induction of TNF is carried with TNF inhibition factor; The NFKp pathway is benchmarked with micronutrients and Gotukola mixture. The TNFp pathway is directly benchmarked with the effects of micronutrients.

**Saturation:** The saturation limits in this pathway are high, since the effect of Gotukola mixture on the NfK and the feedback looks of Cortisol with the effect of micronutrients are predominant, we can observe 100% recovery with TNFa on product formulation.

**
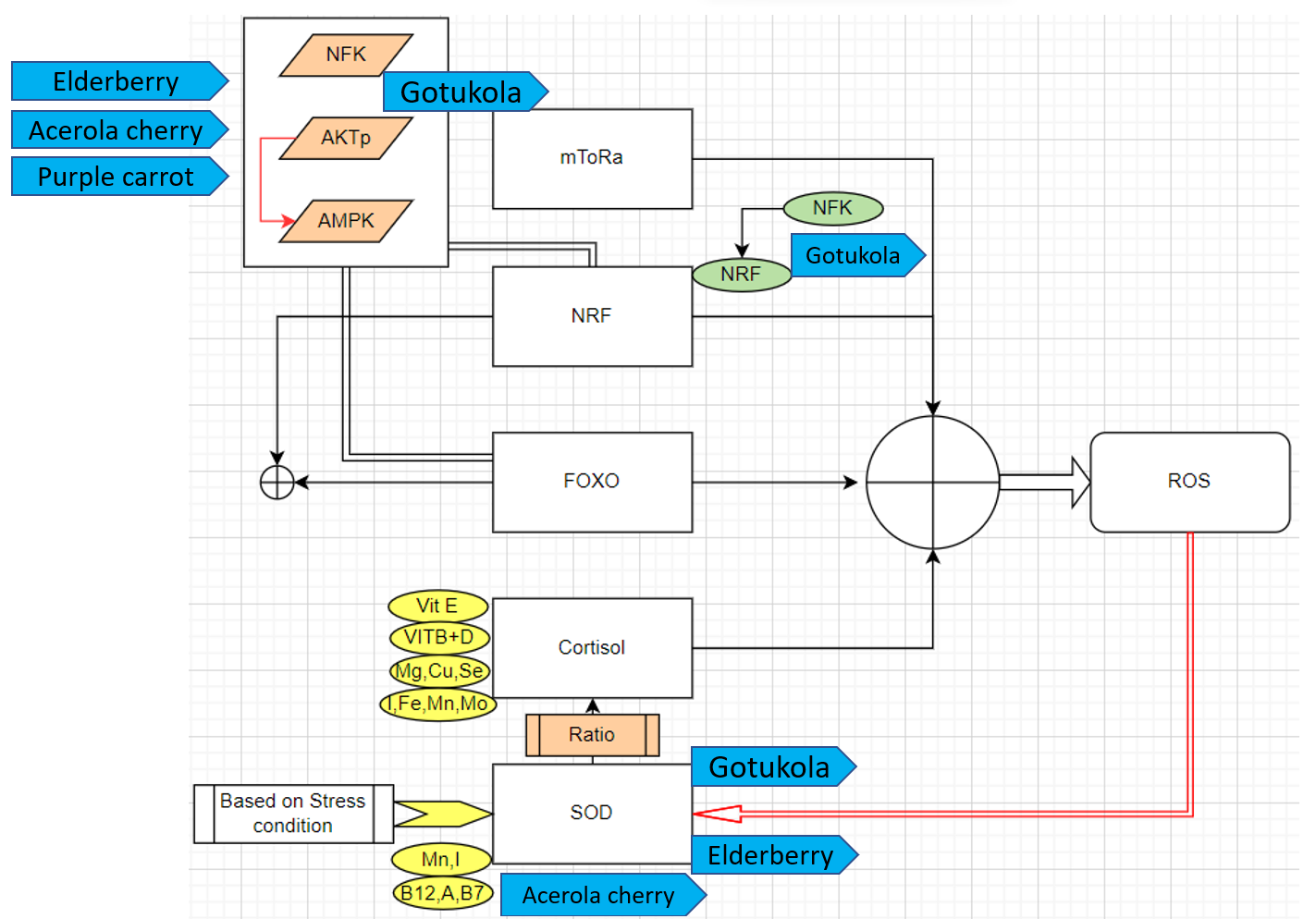
**

**Figure S6: Targeting NRF and Foxo Pathways for Effective Regulation of Oxidative Stress Levels as Assessed by Cortisol to SOD Ratio.**

**Effective pathways:** Cortisol to SOD ratio has been used to induced oxidative stress. The NRF and Foxo pathways are benchmarked to regulate the level of Oxidative stress in addition to induction.

**Saturation:** Cortisol/SOD ratio and activation based on mtora compensate each other, with an upper bound; AKTp saturation on AMPK and NRF as well as FOXO, reaches an upper bound with gotukola. Thus, with complete product the effect reaches a saturation threshold. The effect of micronutrients on Cortisol are lower than effect of Gotukola whereas in SOD this is observed to be inverse; This pushes the saturation limit of the Cortisol/SOD ratio.

**
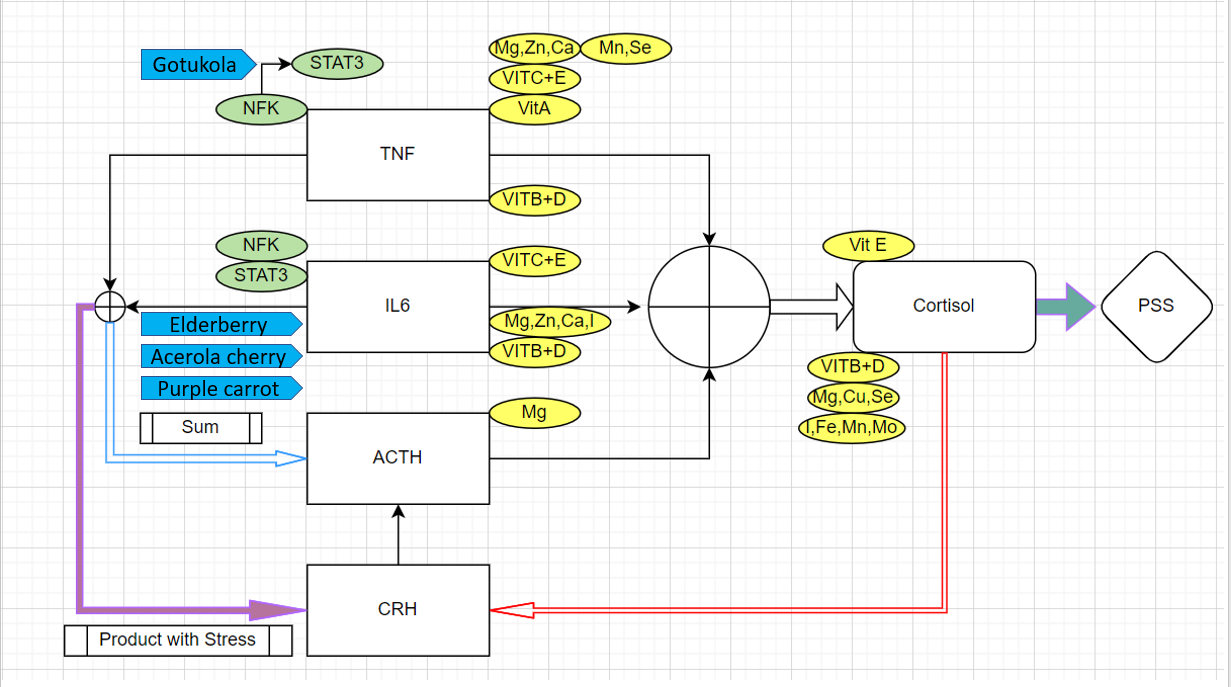
**

**Figure S7: Effective Modulation of Cortisol Levels in Stressful Conditions through Vitamin and Gotukola Mixture Intervention Targeting the ACTH Pathway.**

**Effective pathways**: The stressed condition is induced in cortisol through ACTH; Cortisol increase with stress, which are effectively downregulated with vitamins and Gotukola mixture.

**Saturation:** STAT3p+IL10 are downregulated with Gotukola, but it reaches a saturation as the (TNF*IL6) Stat3p decreased with Vitamins while increased with Gotukola, reaching a saturation point, thus reaching a fixed FC from initial stressed state.

The effect of micronutrients and botanical were modelled by elucidating their target identification and mechanism of action. Zinc's influence on IL6 and TNF-α is modeled by correlating zinc concentration in Daily Plus Nutrilite with the rate of activation of IL6 (‘k25’ parameter) and the activation rate of TNF-α via the NFkb pathway (‘i_k6’ parameter). Similarly, the effect of Vitamin A is explored through its known impact on TNFa, ROS, and IL10. The activation of TNF-α via NF-κB pathway (‘i_k6’ parameter) and rate of Il10 activation via NF-κB pathway (‘i_k7’ parameter) were perturbed to simulate the effect of Vitamin A. Likewise, the impact of Gotukola is incorporated by targeting the activation of STAT3 by NF-κB via rate constant (‘k24a’ parameter) and the rate of Nfkb pathway activation (‘i_k25’ parameter). Similarly, other botanicals and micronutrient effects were modelled to the targets shown in figure S5-S7.

***ODEs***

Insulin Signalling

%Rate of change of insulin in vein

$InsVprime=-(gamma*InsV)+ (((V\_Glu.*(Ca\_Glu .^n./(Ca\_Glu .^n+K\_Glu .^n ))+(V\_Ala.*((Ca\_Ala^na )./(((Ca\_Ala^na )+K\_AA .^na ) )))+(V\_FFA.*(Ca\_FFA .^nf )./((Ca\_FFA .^nf )+K\_FF .^nf )))/(1e-12));$ ----- (1)

%Rate of change of insulin in liver

$InsLprime=(-(m1+((($(-m5*$\left( gamma*InsV \right)$)+ m6$)*m1)/(1-($(-m5*($\left( gamma*InsV \right)$))+ m6$))))*InsL)+(m2*InsP)+ ( gamma*InsV);$ ----- (2)

%Rate of change of insulin in plasma

$InsPprime=\left( -\left( m2+m4 \right)*InsP \right)+\left( m1*InsL \right);$ ----- (3)

%Rate of plasma glucagon formation

$Glnpprime=\left( -\left( a1+a2 \right)*Glnp \right)+\left( \left( \frac{Gm}{1+\left( q1*\exp\left( p1*\left( Ca_{Glu}-5 \right) \right) \right)} \right)+ \left( Vm_{AAg}*\left( \frac{Am_{Glcn}^{nA}}{Am_{Glcn}^{nA}+Km_{AA}^{nA}} \right) \right) \right);$ ---- (4)

%Rate of phosphorylation and dephosphorylation of IR

$insulinp=\left( \left( \frac{InsP}{0.05} \right)*1e-12 \right)*2*\left( \frac{\left( 25e-12 \right)^{2}}{Gnp^{2}+\left( 25e-12 \right)^{2}} \right);$

$IRpprime = \left( 1*k1f*\left( \mathrm{insulinp} \right)*1000*IR + k1aBasic*IR \right)- k1b*IRp;$ ----- (5)

%Rate of phosphorylation and dephosphorylation of IRS

$IRSpprime= \left( k2f + k21f* \left( FB8*\frac{AKTp^{nakt}}{AKTp^{nakt}+kakt^{nakt}} \right)*IRp*IRS \right)-\left( k2b*k_{mul2}*IRSp*\left( 1 + 0.2*\left( \frac{S6K1a^{4}}{6^{4.0}+ S6K1a^{4.0}} \right) \right) \right)$ ----- (6)

%Rate of phosphorylation and dephosphorylation of IRS

$PI3Kpprime = \left( 1*k4f*PI3K*\left( IRSp+i_{k9}*\left( \frac{LPS}{LPS+i_{km8}} \right)+FB3*i_{k21}*\left( \frac{TNf_{nt}^{i}}{TNf_{nt}^{i}+i_{{km17}_{nt}^{î}}} \right)+\left( i_{k21a}*RASa \right) \right) \right)- \left( k4b*PI3Kp \right);$ -----(7)

%Rate of phosphorylation and dephosphorylation of AKT

$AKTpprime =\left( k4f1*AKT*PI3Kp \right)- \left( k_{mul}*k4b1*AKTp \right)$ ----- (8)

%Regulation of mTOR

$mTORaprime = k8f*mTOR*AKTp*\left( \frac{k8f2}{k8f2+AMPK} \right)+k8f1*\left( 1+\left( 2.5*\left( \frac{AA^{ns}}{AA^{ns}+ faa^{ns}} \right) \right) \right)*mTOR + 10*k8f4*mTOR*\left( SFA^{2} \right)+ k8f4*mTOR*\left( UFA^{2} \right)-\left( k8b*mTORa \right);$ ----- (9)

%Rate of phosphorylation and dephosphorylation of S6K1

$S6K1aprime = \left( \left( f20*\left( 3*\left( \frac{PI3K}{4+PI3K} \right) \right)*\left( mTORa \right)*\left( \left( \frac{fto^{nt}}{fto^{nt}+ PP2A^{nt}} \right) \right)*\left( S6K1 \right)*\left( \frac{{0.75}^{2.0}}{{0.75}^{2.0}+AMPK_{Eff}^{2.0}} \right) \right)-\left( f21*\left( PP2Amax*\left( \frac{fpp^{np}}{fpp^{np}+ mTORa^{np}} \right) \right)*\left( S6K1a \right) \right) \right);$ ----- (10)

Inflammatory Signalling

%Regulation of IKK

$IKKpprime = \left( i_{k4}*LPS*IKK*\left( 1-IKK \right) \right)- \left( i_{kd4}*IKKp \right);$ ------ (11)

%Regulation of NfKp

$NfKpprime =\left( \frac{Cortisol}{kcort+Cortisol} \right)*\left( i_{k5}*NfK*\left( 1+i_{k5b}*IKKp \right)*\left( 1+mTORa \right)*\left( 1+FB2*i_{k5a}*TNF \right)*\left( \frac{i_{{ki2}_{n12}^{i}}}{i_{{ki2}_{n12}^{i}}+\left( {il10}_{n12}^{i} \right)} \right)*\left( \frac{i_{ki3a}}{i_{ki3a}+FOXOa} \right) \right)-i_{kd5}*NfKp$;

-------(12)

%Regulation of TNF

$TNFprime=k_{mul4}*\left( i_{k6}*\frac{NfKp_{n6}^{î}}{NfKp_{n6}^{î}+{km12}_{n6}^{î}} \right)- i_{kd6}*TNF$; -------(13)

%Regulation of IL10

$IL10prime= \left( i_{k7}*\frac{NfKp_{n7a}^{i}}{NfKp_{n7a}^{i}+km18 {}_{n7a}^{i}} \right)+\left( \frac{FB6*i_{k7a}*\left( PI3Kp_{n7}^{i} \right)}{\left( km18a_{n7}^{i} \right)+\left( PI3Kp_{n7}^{i} \right)} \right)+i_{k7b}*\left( \frac{STAT3p}{km18b+STAT3p} \right) -i_{kd7}*IL10;$ -------(14)

%Regulation of LPS

$LPSprime = (i\_inf*inf\_load)*(ki10^2/(ki10^2+IL10^2))*(ki10a/(ki10a+FOXOa)) - k\_infd*LPS;$ -------(15)

%Regulation of STAT3p

$STAT3pprime = STAT3*\left( k24a*NfKp*\left( FB7*\left( \frac{IL6}{k24b+IL6} \right) \right)*\left( \frac{k24d}{k24d+FOXOa} \right)*\left( \frac{k24e}{k24e+AMPK} \right)+ \left( \frac{k24c*IL{10}^{2}}{km{24}^{2}+IL{10}^{2}} \right) \right)- kd24*STAT3p;$ -------(16)

%Regulation of IL6

$IL6prime = k_{mul3}*k25a*\left( \frac{STAT3p}{km25+STAT3p} \right)*\left( \frac{NfKp^{2}}{km25a^{2}+NfKp^{2}} \right)- kd25*IL6;$

------(17)

%Regulation of HIF

$HIFpprime = k22*HIF*ROS*mTORa - kd22*HIFp;$ -------(18)

%Regulation of FOXO

$FOXOaprime = k26*FOXO*HIFp*AMPK*\left( \frac{k26a}{k26a+AKTp} \right)- kd26*FOXOa;$ ------(19)

%Regulation of ROS

SOD = 1.8/(1+(a_u));

$ROSprime = \left( 0.05*\frac{Cortisol}{SOD} \right)*k27*\left( \frac{mTORa}{k27c+mTORa} \right)*\left( 1+\left( k_{exer2}*exer \right) \right)*\left( FB1*\left( \frac{k27a}{k27a+FOXOa} \right) \right)*((FB9*k27b)^2/(FB9*k27b^2+NRFa^2))- kd27*ROS;$

-------(20)

%Regulation of AMPK

$AMPKprime=1*\left( kam1*AMP_{ATP_{AMPK}}*\left( AMPKt-AMPK \right) \right)*\left( \frac{kam3}{kam3+HIFp} \right)-Kam2*\left( AMPK \right)*\left( 5*\left( \frac{AKTp^{2}}{AKTp^{2}+{0.05}^{2}} \right) \right);$ -------(21)

%Regulation of AMPK

$NRFaprime= \left( k28*NRF*AKTp*AMPK*\left( \frac{k28a^{kn28}}{k28a^{kn28}+NfKp^{kn28}} \right) \right)- kd28*NRFa;$ -------(22)

HPA Axis

%Regulation of CRH

$dCRH_{dt}= k1*\left( \frac{1}{Cortisol} \right)*\left( \frac{1}{1+\left( \frac{Cortisol}{k_{GR}} \right)^{3}} \right)*TNF*IL6 - w1*CRH;$ --------(23)

%Regulation of ACTH

$dACTH_{dt}= k2*\left( \frac{1}{1+\left( \frac{Cortisol}{k_{GR}} \right)^{3}} \right)*CRH - w2*ACTH + kn_{tnf1}*\left( TNF + IL6 \right);$ -------(24)

%Regulation of Cortisol

$dCortisol_{dt}= 3*k3*ACTH - w3*Cortisol + kn_{tnf2}*\left( TNF + IL6 \right);$ --------(25)

-----------------------Initial Conditions ----------------------

*Initial Conditions*

*IRp_0 = 0; IRSp = 0; b_InsL = 4.4; b_InsP = 1.25; b_InsV = 7.5; Glnp_0 =25e-6; PI3K_0 =10; AKT_0 = 10; mTOR_0 = 10 ; S6K1a_0 =0; IKKp_0 = 0; NfKp_0 = 0; TNF_0 = 0; IL10_0= 0; LPS_0 = 0; STAT3p_0 =0; IL6_0 =0; HIFp_0 =0; FOXOa_0 = 0; ROS_0 = 0; AMPK_0 =0.2; NRFa_0 =0; CRH_0 = 1; ACTH_0 = 1; Cortisol_0 = 1;*

*Parameters*

-----------------------Parameters for Insulin Signalling ----------------------

| kn = 0.378 |
| --- |
| nakt = 0.097 |
| npkc = 0.054 |
| kakt = 0.229 |
| k1aBasic = 7447.29 |
| k1f = 3.640E+11 |
| k1b = 2.93 E+4 |
| k2f = 7.63 |
| k21f = 3472.84 |
| k2b = 1.90990E+04 |
| k4f = 1.2232 E+04 |
| k4b= 8.0754 E+04 |
| k4f1 = 2.7775 E+04 |
| k4b1= 17341.72 |
| k6f = 36142.97 |
| k6b = 1761.31 |
| k5f = 3.4097 E-03 |
| k5b = 11.45 E-03 |
| k8f = 4.6289 E+04 |
| k8f1 = 2.3144 E+04 |
| k8b = 1.15977 E+05 |
| i_k21 = 5.40E-05 |
| i_k9 = 4.81E-05 |
| i_k21a = 5.40E-05 |
| rate of mTOR activation by AKTp and amino acids (AA) |
| k8f2= 0.010 |
| k8f3= 0.54 |
| k8f4= 9257.87 |

| FB3 = 0.054  FB2 = 0.054 |
| --- |
| FFA= FF |
|  |
| mTOR_Raptor activation by AA in absence of insulin |
| AA= Aam |
| ns= 0.16 |
| faa= 0.040 |
|  |
| S6K1 phosphorylation-dephosphorylation |
| PP2Amax = 0.270 |
| AMPK_Eff= 1*AMPK |
| f20= 8.10E-05 |
| f21= 2.7 E-05 |
| fpp= 0.324 |
| fto= 0.108 |
| np= 0.064 |
| nt= 0.216 |
|  |
| -----------------------Parameters for Inflammatory Signalling ---------------------- |
|  |
| i_kd4= 0.00054 |
| i_k4= 0.432 |
| i_k5= 0.0004 |
| i_k5a = 0.0004 |
| i_k5b = 27.02 |
| k5c = 0.0540 |
|  |
| i_ki3 = 0.0540 |
| i_kd5 = 0.009 |
|  |
| i_nt = 0.216 |
| i_n12 = 0.162 |
| i_n7a = 0.108 |
| i_ki3a = 0.27 |
| ki3b = 1.08 |
| i_ki2= 0.54 |
| kisc = 0.54 |
|  |
| FB6 = 0.054 |
| ki10 = 0.054 |
| ki10a = 5.4E-03 |
| i_k7a= 9.0E-04 |
| i_k7c = 2.25E-05 |
| km12= 0.270 |
| i_k6= 0.018 |
| i_kd6= 4.5E-05 |
| i_n6 = 0.162 |
|  |
| i_km18 = 0.32 |
| i_kd7= 9.009E-06 |
| i_k7= 4.5E-03 |
| i_n7=0.108 |
| i_k7b = 4.50E-04 |
| km18a= 5.4E-04 |
| km18b = 0.540 |
| kcort = 0.2  %% Including STAT3, IL6 |
| k23a = 0.010 |
| ki23 = 0.10 |
| kd23 = 8.1E-03 |
| kn23 = 0.135 |
| k24a = 0.027 |
| k24c = 5.4E-04 |
| kd24 = 0.010 |
| k24b = 3.24 |
| k24d = 0.054 |
| k24e = 0.081 |
| km24 = 5.40 |
| k25a = 1.351 |
| ki25 = 0.540 |
| kd25 =5.40E-03 |
| k_exer1 = 0.021 |
| n_SG= 0.251 |
|  |
| km25 = 0.135 |
| km25a = 0.21 |
| %% Including HIF1a, ROS, AMPK |
| k22 = 0.0054 |
| kd22 = 0.010 |
| k26 = 0.027 |
| k26a= 0.10 |
| kd26 = 0.010 |
| k27= 0.540 |
| k27a = 0.081 |
| kd27 = 5.4E-03 |
| k27b = 0.027 |
| k27c= 0.648 |
|  |
| k_exer2 = 5.4E-04 |
|  |
| %% Including NRF2 |
|  |
|  |
| k28 = 0.0540 |
| k28a = 0.0540 |
| kd28 = 5.40E-03 |
| kn28 = 0.108 |
| k29 = 0.043 |
| k29a = 0.0540 |
| k29b = 0.0540 |
| kd29 = 5.40E-03 |
| k30 = 0.0270 |
| k30a = 0.0540 |
| kd30 = 5.40E-03 |
| k31 = 2.7E-03 |
| kd31 = 5.4E-03 |
| ki_alc1 = 0.016 |
| k_alc2 = 0.016 |
|  |
|  |
| %% Inflammasome, autophagy |
|  |
| k33 = 0.5405 |
| kd33 = 5.40E-03 |
| km33 = 0.05405 |
| kn33 = 0.0540 |
| k32 = 1.081 |
| ki32 = 1.08 |
| kd32 = 5.4E-03 |
| km32 = 0.810 |
| km32a = 0.0270 |
| k34 = 5.40E-03 |
| kd34 = 0.010 |
| k35 = 1.081 |
| kd35 = 5.4E-03 |
| km35 = 0.810 |
| k36 = 0.0270 |
| kd36 = 5.4E-03 |
|  |
| AMPKt=1 |
| kam1= 0.054 |
| Kam2= 0.12 |
| kam3 = 0.10 |
|  |
|  |
| % HPA Model |
| % HPA parameters |
| w1= 9.1 E-03 |
| w2= 1.8 E-03 |
| w3= 4.6E-04 |
| k1= 0.0956 |
| k2= 6.8E-03 |
| k3= 7.134E-05 |
| k_GR= 0.216 |
|  |
| % RAAS parameters |
| n_k3= 0.0540 |
| n_kd3= 0.00540 |
| n_k4= 0.054 |
| n_kd4= 0.005 |
| n_k5= 0.0540 |
| n_kd5 = 0.0054 |
| n_k6 = 0.0540 |
| n_kp6 = 0.0540 |
| n_kd6 = 5.41E-03 |
| kn_tnf1 = 5.405 |
| kn_tnf2 = 16.21 |
|  |

u =1 %Stress

k_mul=0. 04

k_mul2=0.11

k_mul3=0.59

k_mul4= 0.2254

| %---Insulin kinetics Parameters-------- |
| --- |
| Vi= 2.7E-03 |
| m1= 0.010 |
| m2= 0.026 |
| m4= 0.0104 |
| m5= 8.21E-05 |
| m6= 0.0349 |
| gamma= 0.0270 |
|  |
| %% Insulin secretion |
| K_AA= 0.032 |
| K_FF= 0.086 |
| na= 0.313 |
| nf= 0.259 |
| n= 0.251 |
| K_Glu= 0.481 |
| V_Glu= 2.59E-12 |
| V_Ala= 1.35E-12 |
| V_FFA= 1.35E-12 |
|  |
| %Gucagon balance--Glcgn |
| Gm= 7.29E-12 |
| p1= 0.0486 |
| q1= 0.540 |
| Ca_Ala-0.25>=0 Am_Glcn= (Ca_Ala-0.25)  Ca_Ala-0.25<0 Am_Glcn= 0 |
| Km_AA= 0.054 |
| nA= 0.24 |
| Vm_AAg= 1.35E-11 |
|  |
| a1= 8.1E-03 |
| a2= 0.016 |


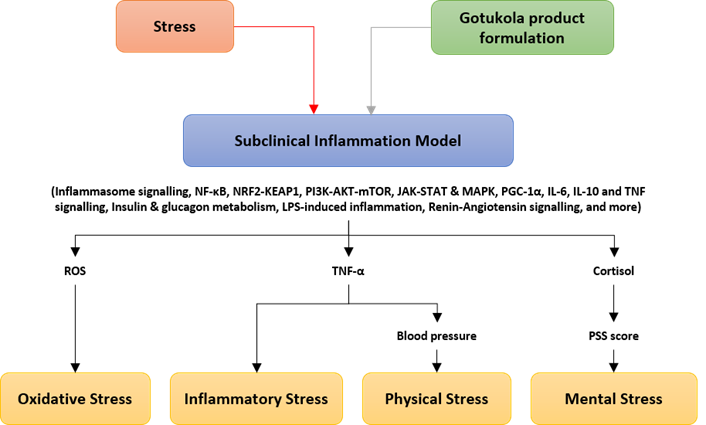


**Figure S8: Schematic representation of the model description**. Input parameters consist of various types of stress and interventions. The stress condition is simulated using a mathematical model that includes various signalling pathways and species (metabolites and cytokines). The output parameters are determined by ROS production, TNF-α, and cortisol levels, which are associated directly/indirectly with oxidative, inflammatory, physical, and mental stress.

**Table S4: The complete list of vitamins, minerals and botanicals used for data mining in this study.**

| **Botanicals** | **Vitamins** | **Minerals** |
| --- | --- | --- |
| Gotu kola ( *Centella asiatica )* | Vitamin A | Calcium |
| Acerola ( *Malpighia emarginata* ) | Vitamin D | Iron |
| Elderberry (*Sambucus* spp.) | Vitamin E | Phosphorus |
| Purple carrot (*Daucus carota* L.) | Vitamin K | Iodine |
|  | Vitamin B1 | Magnesium |
|  | Vitamin B2 | Zinc |
|  | Vitamin B3 | Selenium |
|  | Vitamin B5 | Manganese |
|  | Vitamin B6 | Copper |
|  | Vitamin B7 | Molybdenum |
|  | Vitamin B9 | Chromium |
|  | Vitamin B12 |  |
|  | Vitamin C |  |

**Table S5: Composition of Nutrilite® Daily Plus formulation**

| **Active ingredients** | **UoM** | **Recommended Label Claim** |
| --- | --- | --- |
| Gotukola (*Centella Asiatica* extract)  Equiv. to Gotukola leaf powder 3 g | mg | 120.00 |
| Acerola Cherry | mg | 5.00 |
| Purple Carrot | mg | 5.00 |
| Elderberry | mg | 5.00 |
| Vitamin A (Retinol) | mcg | 840.00 |
| Vitamin D-(Ergocalciferol) | mcg | 15.00 |
| Vitamin E | mg | 10.00 |
| Vitamin K | mcg | 55.00 |
| Vitamin B2 – Riboflavin | mg | 1.90 |
| Dietary folate | mcg | 129.40 |
| Vitamin B12 | mcg | 2.20 |
| Biotin | mcg | 35.00 |
| Iodine | mcg | 140.00 |
| Copper | mg | 1.70 |
| Manganese | mg | 4.00 |
| Molybdenum | mcg | 45.00 |
| Selenium | mcg | 40.00 |
| Zinc | mg | 13.20 |
| Chromium | mcg | 50.00 |
| Iron | mg | 19.00 |
| Vitamin B3 – Niacinamide | mg | 11.00 |
| Vitamin B5 - Pantothenic acid | mg | 5.00 |
| Vitamin B6 – Pyridoxine | mg | 1.90 |
| Vitamin C | mg | 65.00 |
| Vitamin B1- Thiamine | Mg | 1.40 |
| Magnesium | Mg | 100.00 |
| Calcium | Mg | 118.76+31.70 |
| Phosphorus | Mg | 59.88+15.76 |

**References:**

1. Widmer IE, Puder JJ, König C, et al. Cortisol response in relation to the severity of stress and illness. *J Clin Endocrinol Metab*. 2005;90(8):4579-4586. doi:10.1210/jc.2005-0354

2. Ściskalska M, Ołdakowska M, Marek G, Milnerowicz H. Changes in the Activity and Concentration of Superoxide Dismutase Isoenzymes (Cu/Zn SOD, MnSOD) in the Blood of Healthy Subjects and Patients with Acute Pancreatitis. *Antioxidants (Basel, Switzerland)*. 2020;9(10). doi:10.3390/antiox9100948

3. Potashnik R, Bloch-Damti A, Bashan N, Rudich A. IRS1 degradation and increased serine phosphorylation cannot predict the degree of metabolic insulin resistance induced by oxidative stress. *Diabetologia*. 2003;46(5):639-648. doi:10.1007/s00125-003-1097-5

4. Yung HW, Charnock-Jones DS, Burton GJ. Regulation of AKT phosphorylation at Ser473 and Thr308 by endoplasmic reticulum stress modulates substrate specificity in a severity dependent manner. *PLoS One*. 2011;6(3). doi:10.1371/journal.pone.0017894

5. Liu YN, Peng YL, -Liu L, et al. TNFα mediates stress-induced depression by upregulating indoleamine 2,3-dioxygenase in a mouse model of unpredictable chronic mild stress. *Eur Cytokine Netw*. 2015;26(1):15-25. doi:10.1684/ecn.2015.0362

6. Cásedas G, Moliner C, Maggi F, Mazzara E, López V. Evaluation of two different Cannabis sativa L. extracts as antioxidant and neuroprotective agents. *Front Pharmacol*. 2022;13:1009868. doi:10.3389/fphar.2022.1009868

7. González-Ortiz M, Martínez-Abundis E, Robles-Cervantes JA, Ramírez-Ramírez V, Ramos-Zavala MG. Effect of thiamine administration on metabolic profile, cytokines and inflammatory markers in drug-naïve patients with type 2 diabetes. *Eur J Nutr*. 2011;50(2):145-149. doi:10.1007/s00394-010-0123-x

8. Boyali E. Effect of vitamin a supplementation on IFN-γ, TNF-α, IL-2, and IL-6 levels in elite taekwondo players. *Stud Ethno-Medicine*. 2016;10(1):53-58. doi:10.1080/09735070.2016.11905471

9. Huang SC, Wei JCC, Wu DJ, Huang YC. Vitamin B(6) supplementation improves pro-inflammatory responses in patients with rheumatoid arthritis. *Eur J Clin Nutr*. 2010;64(9):1007-1013. doi:10.1038/ejcn.2010.107

10. Mousavi R, Alizadeh M, Asghari Jafarabadi M, et al. Effects of Melatonin and/or Magnesium Supplementation on Biomarkers of Inflammation and Oxidative Stress in Women with Polycystic Ovary Syndrome: a Randomized, Double-Blind, Placebo-Controlled Trial. *Biol Trace Elem Res*. 2022;200(3):1010-1019. doi:10.1007/s12011-021-02725-y

11. Yang B, Gross MD, Fedirko V, McCullough ML, Bostick RM. Effects of calcium supplementation on biomarkers of inflammation and oxidative stress in colorectal adenoma patients: A randomized controlled trial. *Cancer Prev Res*. 2015;8(11):1069-1075. doi:10.1158/1940-6207.CAPR-15-0168

12. Meksawan K, Sermsri U, Chanvorachote P. Zinc supplementation improves anticancer activity of monocytes in type-2 diabetic patients with metabolic syndrome. *Anticancer Res*. 2014;34(1):295-299.

13. Kumari S, Deori M, Elancheran R, Kotoky J, Devi R. In vitro and In vivo Antioxidant, Anti-hyperlipidemic Properties and Chemical Characterization of Centella asiatica (L.) Extract. *Front Pharmacol*. 2016;7:400. doi:10.3389/fphar.2016.00400

14. Intararuchikul T, Teerapattarakan N, Rodsiri R, et al. Effects of Centella asiatica extract on antioxidant status and liver metabolome of rotenone-treated rats using GC–MS. *Biomed Chromatogr*. 2019;33(2):1-9. doi:10.1002/bmc.4395

15. Masola B, Oguntibeju OO, Oyenihi AB. Centella asiatica ameliorates diabetes-induced stress in rat tissues via influences on antioxidants and inflammatory cytokines. *Biomed Pharmacother*. 2018;101:447-457. doi:10.1016/j.biopha.2018.02.115

16. Giribabu N, Karim K, Kilari EK, Nelli S, Salleh N. Oral administration of Centella asiatica (L.) Urb leave aqueous extract ameliorates cerebral oxidative stress, inflammation, and apoptosis in male rats with type-2 diabetes. *Inflammopharmacology*. 2020;28. doi:10.1007/s10787-020-00733-3

17. Choi MJ, Zheng HM, Kim JM, Lee KW, Park YH, Lee DH. Protective effects of Centella asiatica leaf extract on dimethylnitrosamine‑induced liver injury in rats. *Mol Med Rep*. 2016;14(5):4521-4528. doi:10.3892/mmr.2016.5809

18. Hu Y, Yin F, Liu Z, et al. Acerola polysaccharides ameliorate high-fat diet-induced non-alcoholic fatty liver disease through reduction of lipogenesis and improvement of mitochondrial functions in mice. *Food Funct*. 2020;11(1):1037-1048. doi:10.1039/c9fo01611b

19. Oliveira I, Vasconcelos V, Motta V, Da-Silva S. Effects of Oral Vitamin C Supplementation on Anxiety in Students: A Double-Blind, Randomized, Placebo-Controlled Trial. *Pakistan J Biol Sci*. 2015;18:11-18. doi:10.3923/pjbs.2015.11.18

20. Takahashi M, Miyashita M, Park JH, et al. Low-volume exercise training and vitamin E supplementation attenuates oxidative stress in postmenopausal women. *J Nutr Sci Vitaminol (Tokyo)*. 2013;59(5):375-383. doi:10.3177/jnsv.59.375

21. Nussbaumerova B, Rosolova H, Krizek M, et al. Chromium Supplementation Reduces Resting Heart Rate in Patients with Metabolic Syndrome and Impaired Glucose Tolerance. *Biol Trace Elem Res*. 2018;183(2):192-199. doi:10.1007/s12011-017-1128-6

22. Yanni AE, Kokkinos A, Psychogiou G, et al. Daily consumption of fruit-flavored yoghurt enriched with vitamins B contributes to lower energy intake and body weight reduction, in type 2 diabetic patients: a randomized clinical trial. *Food Funct*. 2019;10(11):7435-7443. doi:10.1039/c9fo01796h

23. Foroozanfard F, Jamilian M, Bahmani F, et al. Calcium plus vitamin D supplementation influences biomarkers of inflammation and oxidative stress in overweight and vitamin D-deficient women with polycystic ovary syndrome: A randomized double-blind placebo-controlled clinical trial. *Clin Endocrinol (Oxf)*. 2015;83(6):888-894. doi:10.1111/cen.12840

24. Khoshfetrat MR, Mohammadi F, Mortazavi S, et al. The effect of iron-vitamin C co-supplementation on biomarkers of oxidative stress in iron-deficient female youth. *Biol Trace Elem Res*. 2013;153(1-3):171-177. doi:10.1007/s12011-013-9695-7

25. Farrokhian A, Bahmani F, Taghizadeh M, et al. Selenium Supplementation Affects Insulin Resistance and Serum hs-CRP in Patients with Type 2 Diabetes and Coronary Heart Disease. *Horm Metab Res = Horm und Stoffwechselforsch = Horm Metab*. 2016;48(4):263-268. doi:10.1055/s-0035-1569276

26. Mairuae N, Cheepsunthorn P, Buranrat B. Anti-inflammatory and anti-oxidative effects of Centella asiatica extract in lipopolysaccharide-stimulated BV2 microglial cells. *Pharmacogn Mag*. 2019;14:140. doi:10.4103/pm.pm_197_18

27. Alvarez-Suarez JM, Giampieri F, Gasparrini M, et al. The protective effect of acerola (Malpighia emarginata) against oxidative damage in human dermal fibroblasts through the improvement of antioxidant enzyme activity and mitochondrial functionality. *Food Funct*. 2017;8(9):3250-3258. doi:10.1039/c7fo00859g

28. Abiri B, Vafa M, Azizi-Soleiman F, et al. Changes in Bone Turnover, Inflammatory, Oxidative Stress, and Metabolic Markers in Women Consuming Iron plus Vitamin D Supplements: a Randomized Clinical Trial. *Biol Trace Elem Res*. 2021;199(7):2590-2601. doi:10.1007/s12011-020-02400-8

29. Saad-Hussein A, Moubarz G, Mohgah SA, Wafaa GS, Aya HM. Role of antioxidant supplementation in oxidant/antioxidant status and hepatotoxic effects due to aflatoxin B1 in wheat miller workers. *J Complement Integr Med*. 2019;16(4):1-7. doi:10.1515/jcim-2018-0218

30. Ramezani A, Yousefinejad A, Javanbakht MH, Derakhshanian H, Tahbaz F. Effect of beta-carotene enriched carrot juice on inflammatory status and fasting blood glucose in type 2 diabetic patients. *Curr Top Nutraceutical Res*. 2014;12(1-2):1-8.

31. T. C, P. L, P. C, T. L, M. S, J. L. Serum levels of IL-6 and TNF-alpha in chronic hepatitis b-induced child-pugh B cirrhosis patients after additional treatment of vitamins A and C and their value in evaluation of prognosis. *Int J Clin Exp Med*. 2019;12(4):3758-3765. http://www.ijcem.com/files/ijcem0088880.pdf%0Ahttp://ovidsp.ovid.com/ovidweb.cgi?T=JS&PAGE=reference&D=emed20&NEWS=N&AN=2001970489

32. de Moura MSB, Soares NRM, Barros SÉ de L, et al. Zinc gluconate supplementation impacts the clinical improvement in patients with ulcerative colitis. *BioMetals*. 2020;33(1):15-27. doi:10.1007/s10534-019-00225-0
